# WDR4 promotes the progression and lymphatic metastasis of bladder cancer via transcriptional down-regulation of ARRB2

**DOI:** 10.1038/s41389-023-00493-z

**Published:** 2023-10-02

**Authors:** Guoli Wang, Xin He, Huiqi Dai, Lingyi Lin, Wenmin Cao, Yao Fu, Wenli Diao, Meng Ding, Qing Zhang, Wei Chen, Hongqian Guo

**Affiliations:** 1 Department of Urology, Nanjing Drum Tower Hospital, Clinical College of Nanjing University of Chinese Medicine, Nanjing, 210008 China; 2 Department of Urology, Nanjing Drum Tower Hospital, The Affiliated Hospital of Nanjing University Medical School, Institute of Urology, Nanjing University, Nanjing, 210008 China; 3 Department of Pathology, Nanjing Drum Tower Hospital, The Affiliated Hospital of Nanjing University Medical School, Nanjing, Jiangsu China

**Keywords:** Tumour biomarkers, Bladder cancer

## Abstract

Lymph node (LN) metastasis is one of the key prognostic factors in bladder cancer, but its underlying mechanisms remain unclear. Here, we found that elevated expression of WD repeat domain 4 (WDR4) in bladder cancer correlated with worse prognosis. WDR4 can promote the LN metastasis and proliferation of bladder cancer cells. Mechanistic studies showed that WDR4 can promote the nuclear localization of DEAD-box helicase 20 (DDX20) and act as an adaptor to bind DDX20 and Early growth response 1 (Egr1), thereby inhibiting Egr1-promoted transcriptional expression of arrestin beta 2 (ARRB2) and ultimately contributing to the progression of bladder cancer. Immunohistochemical analysis confirmed that WDR4 expression is also an independent predictor of LN metastasis in bladder cancer. Our results reveal a novel mechanism of LN metastasis and progression in bladder cancer and identify WDR4 as a potential therapeutic target for metastatic bladder cancer.

## Introduction

There are two types of bladder cancer: non-muscle-invasive bladder cancer (NMIBC) and muscle-invasive bladder cancer (MIBC). MIBC tends to progress, and the primary type of metastasis is lymph node (LN) metastasis [1]. Cancer cells can spread from the bladder to pelvic LNs and later to other organs [2]. LN metastasis is believed to be a major cause of poor prognosis in patients with bladder cancer. Approximately 30% of MIBC patients have developed distant metastases at the time of diagnosis. Mortality is significantly higher in bladder cancer patients with LN metastasis, who have a 5-year survival rate of 18.6% [3–5]. Once bladder cancer patients develop LN metastasis, available treatment options are limited, and neither chemotherapy nor radical cystectomy can significantly improve the prognosis [3, 6]. Therefore, accurate and effective treatments for bladder cancer and LN metastasis need to be developed.

Targeted therapies for various cancers have been developed rapidly in recent years, but those for bladder cancer are still in the initial stage of development [7, 8]. An understanding of the molecular mechanisms of bladder cancer progression and LN metastasis may provide valuable therapeutic targets for clinical intervention. It has been reported that vascular endothelial growth factor C (VEGF-C) can promote the formation of lymphatic vessels, and anti-VEGF-C antibodies can inhibit LN metastasis [9]. LN metastasis-associated transcript 1 (LNMAT1) promotes LN metastasis by upregulating CCL2 to recruit macrophages to secrete VEGF-C [10]. Recently, bladder cancer cells have also been found to induce lymphangiogenesis and metastasis independent of VEGF-C by enhancing PROX1 transcription [11]. However, previous studies of LN metastasis in bladder cancer have mainly focused on lymphatic vessel formation and the tumor microenvironment, while there has been limited work on the properties of proteins in bladder cancer cells and their role in LN metastasis.

Members of the WD repeat family are involved in numerous different cellular processes, including cell cycle progression, signal transduction, and gene expression regulation. WD repeat domain 4 (WDR4), a member of the WD repeat protein family, has various functions in different tumors. WDR4 forms a methyltransferase complex with Methyl transferase-like protein 1 (METTL1), promoting the translation of tumor-related mRNA by influencing m7G modification of tRNA [12–15]. In hepatocellular carcinoma, WDR4 interacts with the translation initiation factor EIF2A to promote the translation of CCNB1 and thus promote proliferation, metastasis and sorafenib resistance [16]. WDR4 can also act as an adaptor and mediate the ubiquitination-mediated degradation of the tumor suppressor gene PML, thus promoting the progression of lung cancer [17]. A pancancer study revealed that WDR4 is associated with the prognosis of bladder cancer [18]. More intriguingly, the structure of WD repeat family proteins provides a foundation for scaffold protein interactions, and many can be targeted by small molecule drugs [19]. However, the role of WDR4 in bladder cancer remains unclear.

In this study, WDR4 was investigated and found to be highly expressed in bladder cancer tissue by protein profiling. We found that WDR4 promotes the metastasis and proliferation of bladder cancer cells. The results of mechanistic studies suggested that WDR4 can recruit DEAD-box helicase 20 (DDX20) into the nucleus and inhibit the transcriptional expression of arrestin beta 2 (ARRB2), thus promoting LN metastasis and progression of bladder cancer.

## Results

### WDR4 is associated with bladder cancer progression

**Fig. 1:**
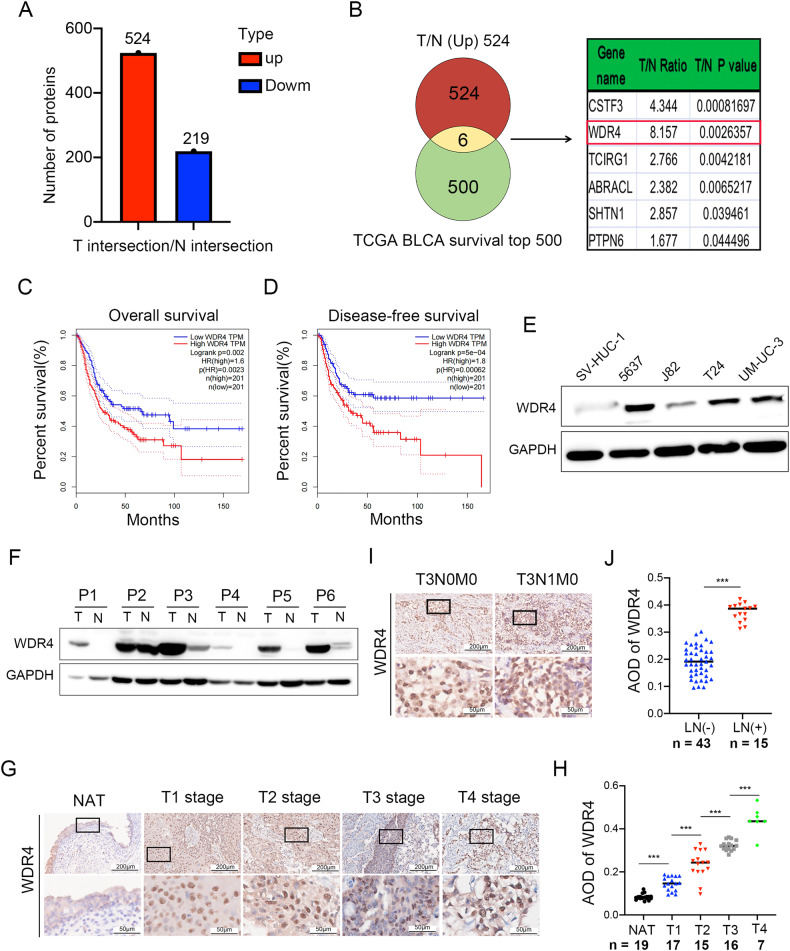
Increased expression of WDR4 correlates with progression and survival in bladder cancer. **A** Label-free quantitative proteomic analysis of cancer tissues (T) and paired normal adjacent tissues (N) from 17 MIBC patients. A total of 524 genes with higher expression and 219 genes with lower expression in cancer tissues (_log2_ FC > 1.5, *p* < 0.05) were identified. **B** Venn diagram analysis of these 524 highly expressed genes and the top 500 genes affecting bladder cancer prognosis in the TCGA database. The table on the right shows the fold changes in the expression of the 6 genes present in both sets. Kaplan‒Meier analysis of overall survival (**C**) and disease-free survival (**D**) in bladder cancer patients in the TCGA cohort with low or high levels of WDR4. **E** WDR4 upregulation in bladder cancer cells and the control cell line SV-HUC-1 was verified by WB. **F** Western blot analysis of the WDR4 protein level in 6 pairs of human clinical bladder cancer samples. **G**, **H** Representative IHC images of WDR4 expression in T1-T4 stage cancer tissues and normal adjacent tissues (NATs) from bladder cancer patients. **I**, **J** Representative IHC images of WDR4 expression in bladder cancer tissues from patients with or without LN metastasis. Scale bar of upper panel = 200 μm, Scale bar of lower panel = 50 μm.

To clarify the differentially expressed proteins that drive malignant progression, tumor tissues (T) and normal tissues (N) from 17 MIBC patients were analyzed by label-free quantitative proteomics. We compared protein levels in these tissues and identified 524 proteins highly expressed in cancer tissues (Log_2_ FC > 1.5, *p* < 0.05) (Fig. 1A, Supplementary Table S1). In addition, in the TCGA database, we identified the top 500 genes that affect the prognosis of bladder cancer (Supplementary Table S2). Only 6 genes were present in both sets of data (Fig. 1B). One of them, WDR4, was associated with poor prognosis and showed much more prominent elevation in bladder cancer tissues than did the other 5 overlapping genes (Fig. 1B–D). Moreover, WDR4 had the most significant effect on overall survival (OS) and disease-free survival (DFS) (Fig. 1C, D, Supplementary Fig. S1). These data suggested that WDR4 may be associated with the malignant progression of bladder cancer.

We next validated the expression of WDR4 in bladder cancer cells and clinical samples. Higher WDR4 levels were measured in bladder cancer cells and tissues (Fig. 1E, F). This finding was further strengthened by IHC analysis. WDR4 was upregulated in tumor tissues compared to normal adjacent tissues, and there was a correlation between the intensity of WDR4 staining and T stage (Fig. 1G, H). Moreover, higher WDR4 expression was observed in metastatic cancer tissues than in nonmetastatic cancer tissues of patients (Fig. 1I, J). Cytoplasmic WDR4 has been reported to play different roles in tumors by regulating the translation or degradation of proteins [20]. Notably, WDR4 was upregulated primarily in the nucleus (Fig. 1G–J). WDR4 expression in tumor cell nuclei has not been reported thus far. Together, these findings indicate that high expression of WDR4 is associated with the progression and metastasis of bladder cancer, probably in a manner related to the nuclear function of WDR4.

### WDR4 promotes LN metastasis of bladder cancer

**Fig. 2:**
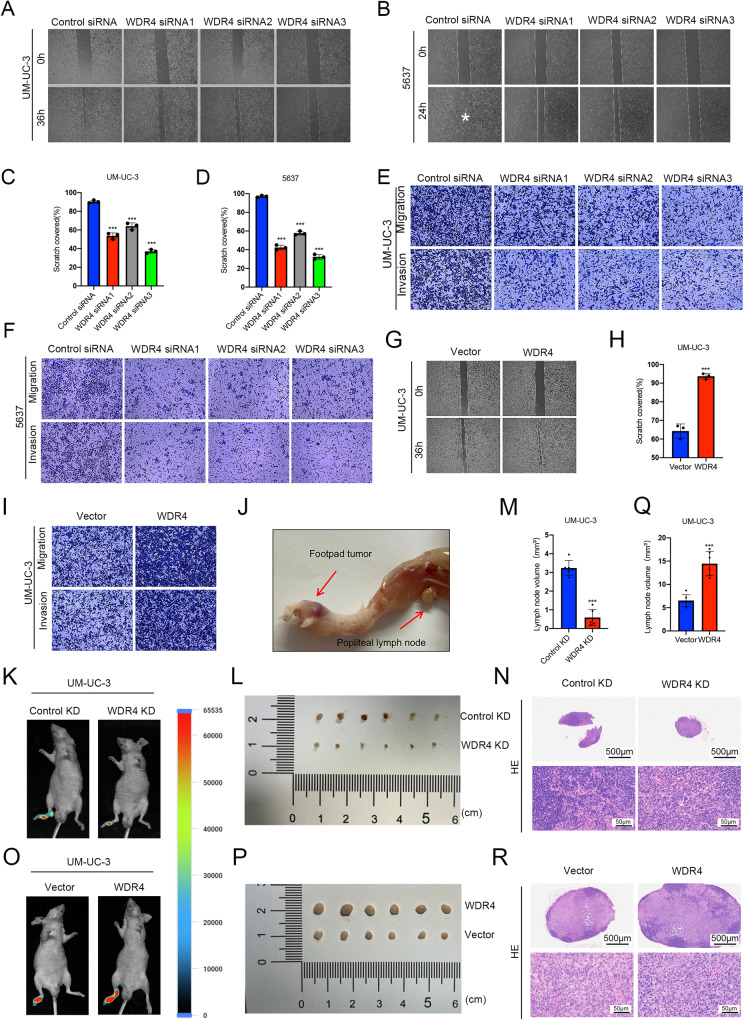
WDR4 promotes the metastasis of bladder cancer cells in vitro and in vivo. **A**–**F** Cells were transfected with WDR4-specific siRNAs or the control siRNA. Interference with WDR4 expression significantly reduced the migration of UM-UC-3 (**A**, **C**) and 5637 (**B**, **D**) cells in the wound healing assay and the migration and invasion of UM-UC-3 (**E**) and 5637 (**F**) cells in the Transwell assays. WDR4 overexpression promoted the migration of UM-UC-3 cells in the wound healing assay (**G**, **H**) and migration and invasion in the Transwell assays (**I**). **J** Representative image of the popliteal LN metastasis mouse model. **K** Representative bioluminescence images of mice with LN metastases generated by WDR4 knockdown (WDR4 KD) and control knockdown (Control KD) UM-UC-3 cells. Representative image of excised popliteal LNs (**L**) and histogram of LN volumes in control KD and WDR4 KD mice (*n* = 6 per group) (**M**). **N** Hematoxylin-eosin (HE) staining of LNs from control KD and WDR4 KD mice. **O** Representative bioluminescence images of mice with LN metastases generated by WDR4 overexpression (WDR4) and control (Vector) UM-UC-3 cells. Representative image of excised popliteal LNs (**P**) and histogram of LN volumes in the vector and WDR4 groups (*n* = 6 per group) (**Q**). The data are presented as the means ± SDs, ***p < 0.001. **R** HE staining of LNs from the control vector and WDR4 overexpression groups. Scale bar of upper panel = 500 μm, Scale bar of lower panel = 50 μm.

We investigated the effect of WDR4 on the cellular processes of migration and invasion. The expression of WDR4 was transiently silenced with three independent siRNAs in UM-UC-3 and 5637 cells (Supplementary Fig. S2A–D). The wound healing assay results showed that silencing WDR4 reduced cell migration (Fig. 2A–D). The results of Transwell assays also demonstrated that transient silencing of WDR4 significantly inhibited cell migration and invasion (Fig. 2E, F). Stable knockdown of WDR4 significantly suppressed cell migration and invasion (Supplementary Fig. S2E, G). In addition, overexpression of WDR4 increased the migration and invasion abilities of bladder cancer cells (Fig. 2G–I, Supplementary Fig. S2F).

LN metastasis is the main type of bladder cancer metastasis [1]. The above data suggested that WDR4 may contribute to lymphatic metastasis of bladder cancer. For further validation, we constructed a popliteal LN metastasis model, which simulated the lymphatic metastasis of bladder cancer in vivo (Fig. 2J). We stably knocked down WDR4 and overexpressed WDR4 in UM-UC-3 cells (Fig. S2E, F). To test their metastatic potential, these cells were inoculated into the footpads of nude mice. As revealed by measurement of luminescence intensity, knockdown of WDR4 significantly suppressed lymphatic metastasis (Fig. 2K), whereas overexpression of WDR4 markedly accelerated the metastasis of bladder cancer cells to LNs (Fig. 2O). The volume of popliteal LNs was significantly smaller in the WDR4-knockdown group (Fig. 2L, M) but significantly larger in the WDR4-overexpression group than in the control group (Fig. 2P–Q). These findings were validated by hematoxylin-eosin (HE) staining of lymphatic metastases (Fig. 2N, R). Together, these results showed that WDR4 promotes LN metastasis in bladder cancer.

### WDR4 promotes the proliferation of bladder cancer cells

**Fig. 3:**
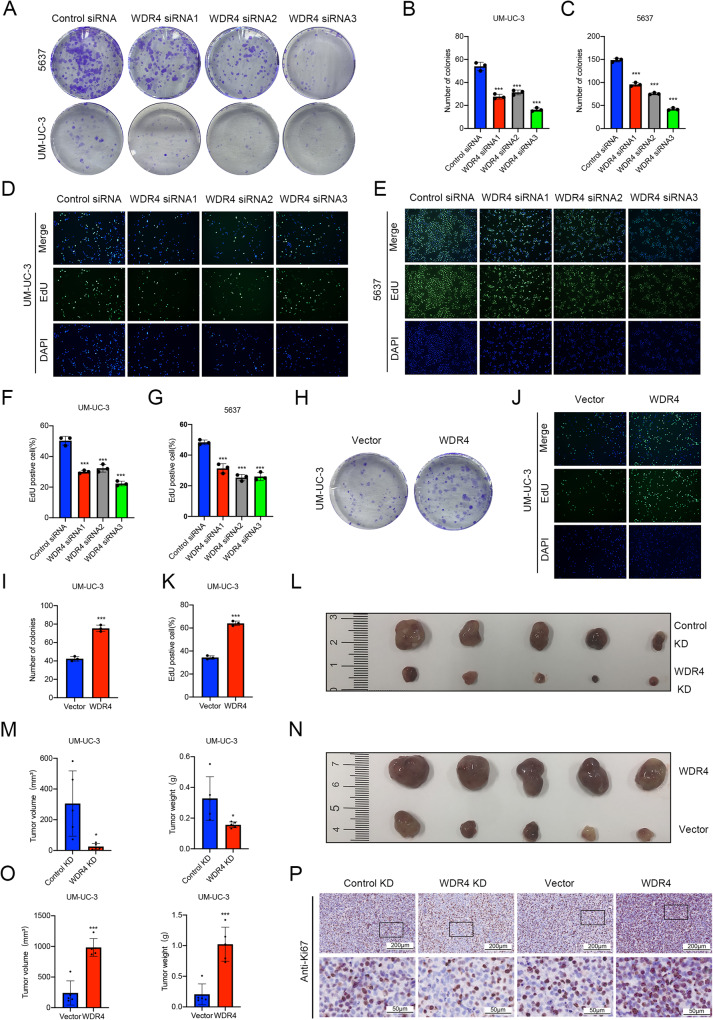
WDR4 promotes the proliferation of bladder cancer cells in vitro and in vivo. **A**–**G** Cells were transfected with WDR4-specific siRNAs and the corresponding control siRNA. Interference with WDR4 expression significantly reduced the numbers of UM-UC-3 (**A**, **B**) and 5637 (**A**, **C**) cell colonies in the colony formation assay and proliferating UM-UC-3 (**D**, **F**) and 5637 (**E**, **G**) cells in the EdU incorporation assay. WDR4 overexpression increased the number of UM-UC-3 cell colonies in the colony formation assay (**H**, **I**) and proliferating UM-UC-3 cells in the EdU incorporation assay (**J**, **K**). Representative image of subcutaneous tumors in WDR4 KD and control KD mice (**L**). Histogram showing the tumor volumes and weights (**M**). Representative image of the tumors in the WDR4 overexpression and control vector groups (**N**). Histogram showing the tumor volumes and weights (**O**). The data are presented as the means ± SDs, *p < 0.05, ***p < 0.001. **P** IHC staining showed the difference in Ki67 expression in tumor samples, Scale bar of upper panel = 200 μm, Scale bar of lower panel = 50 μm.

The ability to proliferate is also a vital intrinsic property of metastatic cancer cells [21]. We next investigated the effect of WDR4 on the cellular proliferation process. Transient silencing of WDR4 significantly reduced cell proliferation (Fig. 3A–G). Stable knockdown of WDR4 also significantly suppressed cell proliferation (Supplementary Fig. S2H). Moreover, overexpression of WDR4 obviously increased the proliferation of bladder cancer cells (Fig. 3H–K).

UM-UC-3 cells with stable WDR4 knockdown or WDR4 overexpression and the corresponding control cells were subcutaneously inoculated into nude mice (Supplementary Fig. S3). Knockdown of WDR4 significantly decreased tumor volume and weight (Fig. 3L, M), while overexpression of WDR4 markedly increased the volumes and weights of the tumors (Fig. 3N, O). Furthermore, immunohistochemical (IHC) analysis of xenograft tumors confirmed that WDR4 knockdown resulted in lower levels of Ki67 expression, but WDR4 overexpression resulted in higher levels of Ki67 expression (Fig. 3P). These results revealed that WDR4 promotes the proliferation of bladder cancer cells.

### DDX20 interacts with WDR4

**Fig. 4:**
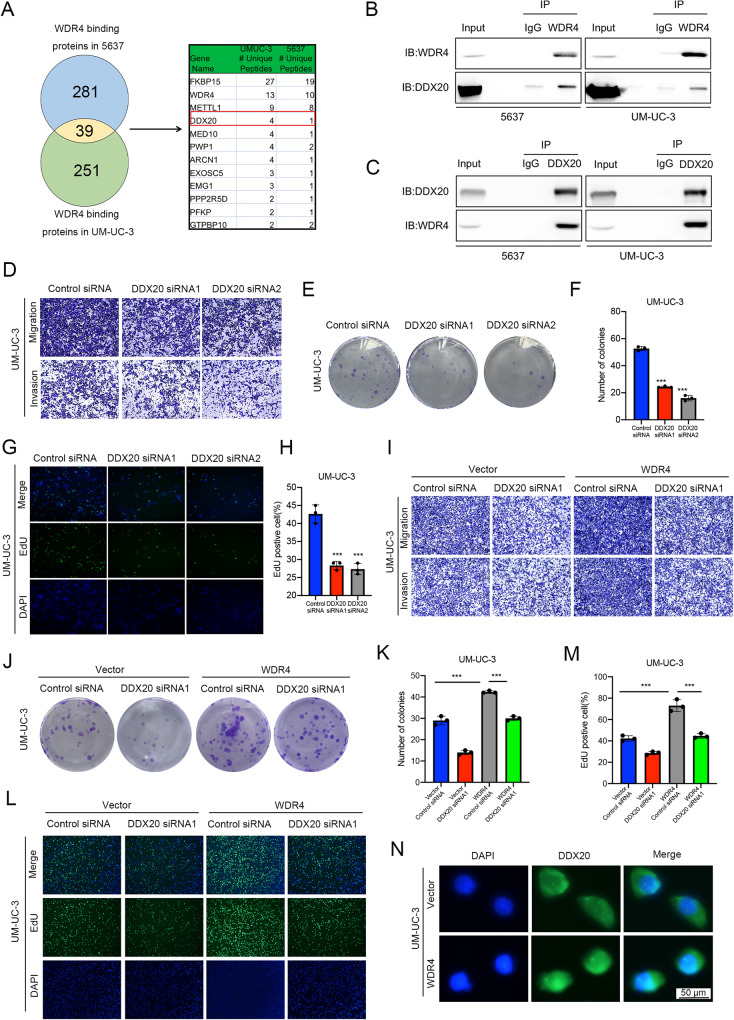
DDX20 interacts with WDR4 and promotes the metastasis and proliferation of bladder cancer cells. **A** The interacting proteins were immunoprecipitated with an anti-WDR4 antibody. Venn diagram showing the 251 and 281 proteins identified in UM-UC-3 and 5637 cells, respectively, using LC‒MS/MS. The table on the right shows the top 12 proteins to which WDR4 binds in both cell lines. **B**, **C** Co-IP and Western blot analyses showed the interaction between endogenous WDR4 and DDX20 in UM-UC-3 and 5637 cells. **D** Cells were transfected with DDX20-specific siRNAs and the corresponding control siRNA. Interference with DDX20 expression significantly reduced the migration and invasion of UM-UC-3 cells in the Transwell assays. Interference with DDX20 expression significantly reduced the numbers of UM-UC-3 cell colonies in the colony formation assay (**E**, **F**) and proliferating UM-UC-3 cells in the EdU incorporation assay (**G**, **H**). **I**–**M** WDR4 overexpression and control vector UM-UC-3 cells were transfected with DDX20-specific siRNAs and the corresponding control siRNA. Migration and invasion of cells were evaluated using Transwell assays. **I** A colony formation assay (**J**, **K**) and a EdU incorporation assay (**L**, **M**) were used to evaluate the cell proliferation ability. The data are presented as the means ± SDs, ***p < 0.001. **N** The location of DDX20 was determined in WDR4-overexpressing and control UM-UC-3 cells by using IF staining, Scale bar 50 μm.

WDR4 commonly binds to specific proteins to perform its function [14]. To investigate how WDR4 regulates bladder cancer progression, we performed co-IP experiments on bladder cancer cells. Intracellular proteins that could interact with WDR4 were immunoprecipitated with specific antibodies from UM-UC-3 and 5637 cells and then explored using LC‒MS/MS analysis. A total of 251 and 281 proteins were identified in UM-UC-3 and 5637 cells, respectively. Of these proteins, 39 were present in both sets (Fig. 4A). WDR4 forms a heterodimer with METTL1 [22]. METTL1 ranked high on the list of proteins that might interact with WDR4 (Fig. 4A, Supplementary Table S3). The METTL1-WDR4 complex has been found to promote cell transformation and tumor progression in bladder cancer [23, 24]. Notably, WDR4 was found to be enriched in the nucleus in bladder cancer cells (Fig. 1G–J), and we explored its mechanism and function in the nucleus. DEAD-box helicase 20 (DDX20), a member of the DEAD-box helicase family, also ranked high on the list of proteins that may interact with WDR4 (Fig. 4A, Supplementary Table S3). In recent years, the DEAD-box helicase family has been found to be involved in transcriptional regulation in the nucleus [25]. The results of co-IP experiments confirmed the interaction between WDR4 and DDX20 (Fig. 4B, C). To determine whether DDX20 contributes to bladder cancer cell metastasis and proliferation, we knocked down DDX20 in UM-UC-3 cells (Fig. S4A). Knockdown of DDX20 significantly suppressed cell migration, invasion and proliferation (Fig. 4D–H). Subsequently, we studied the cooperative role of WDR4 and DDX20 in bladder cancer cells. Compared with that in the control group, WDR4 overexpression promoted the metastasis and proliferation of cancer cells, but simultaneously silencing DDX20 partially reversed the increases in the metastasis and proliferation of bladder cancer cells caused by WDR4 upregulation (Fig. 4I–M). Surprisingly, overexpression of WDR4 promoted the nuclear accumulation of DDX20 (Fig. 4N). Thus, WDR4 may interact with DDX20 in the nucleus to promote the progression of bladder cancer.

### ARRB2 is repressed at the transcriptional level by WDR4 and DDX20

**Fig. 5:**
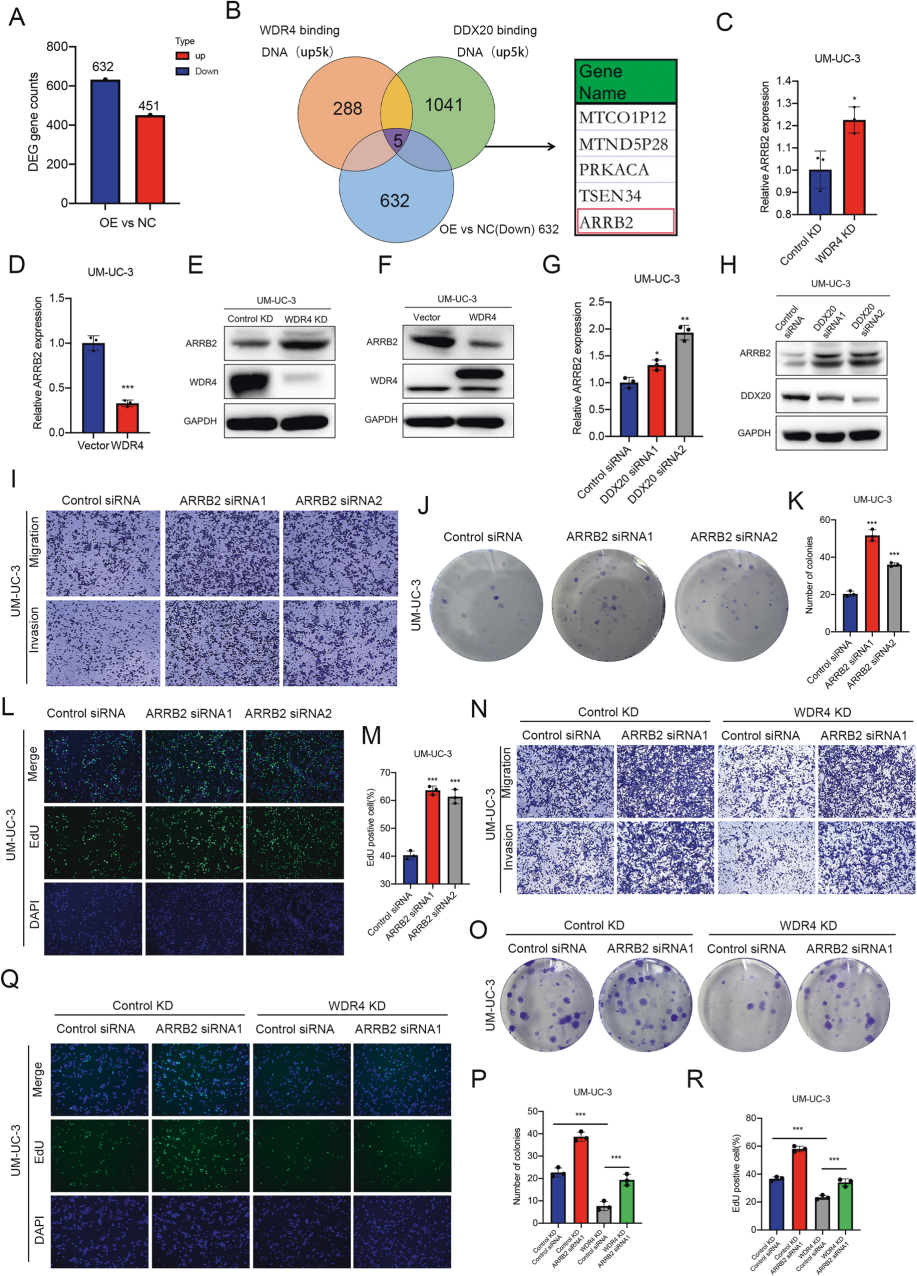
WDR4 and DDX20 inhibit ARRB2 expression. **A** RNA sequencing analysis of UM-UC-3 cells with stable WDR4 overexpression cells and control UM-UC-3 cells. A total of 451 genes with higher expression and 632 genes with lower expression in WDR4-overexpressing cells were identified. **B** CUT&Tag-seq was used to identify the transcriptional regulatory regions to which WDR4 and DDX20 proteins bound. The Venn diagram shows the number of genes bound by WDR4 and DDX20 and the number of genes downregulated after WDR4 overexpression in UM-UC-3 cells. The table on the right shows the 5 genes present in all three datasets. The mRNA levels of ARRB2 in WDR4 KD and control KD cells (**C**) and in WDR4 overexpression (WDR4) and control (Vector) cells (**D**) were measured by RT‒qPCR. The protein levels of ARRB2 in WDR4 KD and control KD cells (**E**) and WDR4 overexpression (WDR4) and control (Vector) cells (**F**) were measured by WB. UM-UC-3 cells were transfected with DDX20-specific siRNAs and the corresponding control siRNA. The mRNA levels of ARRB2 were measured by RT‒qPCR (**G**). The protein levels of ARRB2 were measured by WB (**H**). **I** Cells were transfected with ARRB2-specific siRNAs and the corresponding control siRNA. Interference with ARRB2 expression significantly increased the migration and invasion of UM-UC-3 cells in the Transwell assays. Interference with ARRB2 expression significantly increased the numbers of UM-UC-3 cell colonies in the colony formation assay (**J**, **K**) and proliferating UM-UC-3 cells in the EdU incorporation assay (**L**, **M**). **N** WDR4 KD and Control KD UM-UC-3 cells were transfected with ARRB2-specific siRNAs and the corresponding control siRNA. Migration and invasion of cells were evaluated using Transwell assays. A colony formation assay (**O**, **P**) and an EdU incorporation assay (**Q**, **R**) were used to evaluate cell proliferation. The data are presented as the means ± SDs, *p < 0.05, **p < 0.01, ***p < 0.001.

WDR4 has been reported to regulate translation by interacting with proteins in the cytosol. However, we found that the nuclear expression was significantly higher than cytoplasmic expression of WDR4 in bladder cancer cells (Fig. 1G–J). In addition, the interacting protein DDX20 has been reported to act as a repressor of gene transcription [26–29]. The WDR4-DDX20 complex may affect the progression of bladder cancer by regulating the transcription of target genes. To identify the key molecules regulated by the complex, CUT&Tag-seq was performed to identify the respective binding DNA locations of WDR4 and DDX20 (Fig. S4B, C). Moreover, RNA-seq was performed on cells stably overexpressing WDR4 and the corresponding control cells to screen for the differentially expressed genes (Fig. 5A). The numbers of genes with downregulated mRNA expression after WDR4 overexpression, genes encoding proteins binding to WDR4 and genes encoding proteins binding to DDX20 in transcriptional regulatory regions were 632, 288 and 1041, respectively (Fig. 5B and Supplementary Tables S4–6). There were 5 genes (MTCO1P12, MTND5P28, PRKACA, TSEN34, ARRB2) present in all three sets (Fig. 5B). In addition, ARRB2 has been reported to inhibit the growth and progression of bladder cancer and increase the response to chemotherapy [30]. Both WDR4 and DDX20 can bind the transcriptional regulatory region of ARRB2 (Fig. S4D). ARRB2 may act as a tumor suppressor regulated by the WDR4-DDX20 complex in bladder cancer. Next, knockdown of WDR4 was found to increase the expression of ARRB2 (Fig. 5C, E, Supplementary Fig. S3), whereas overexpression of WDR4 in cells markedly suppressed the expression of ARRB2 (Fig. 5D, F, Supplementary Fig. S3). In addition, transient knockdown of DDX20 increased the expression of ARRB2 in cells (Fig. 5G, H). Moreover, we observed that ARRB2 knockdown enhanced cell migration, invasion and proliferation (Fig. 5I–M, Supplementary Fig. S4G–H). Together, these results confirmed that ARRB2 may be transcriptionally regulated by WDR4 and DDX20. Subsequently, we examined whether ARRB2 could reverse the effects of WDR4 in bladder cancer cells. WDR4 knockdown inhibited the metastasis and proliferation of cancer cells compared with that of the control cells, and simultaneous silencing of ARRB2 significantly restored the metastasis and proliferation of bladder cancer cells (Fig. 5N–R).

### The WDR4-DDX20 complex inhibits Egr1-promoted transcription of ARRB2

**Fig. 6:**
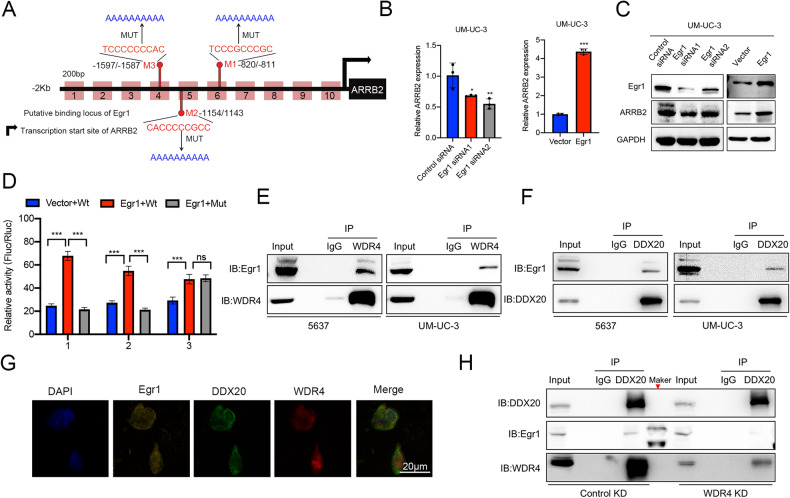
WDR4 regulates bladder cancer progression by transcriptional repression of ARRB2. **A** Egr1-binding sites in the promoter of the ARRB2 gene; the three corresponding mutation sites are marked with red vertical lines. **B** After Egr1 knockdown or Egr1 overexpression in UM-UC-3 cells, the mRNA levels of ARRB2 were measured by RT‒qPCR. **C** The protein levels of ARRB2 were measured by WB. **D** The Egr1 overexpressing plasmid and the reporter plasmids containing the wild-type (WT) or mutated (MUT) of Egr1 binding sequence in the ARRB2 promoter were cotransfected into 293 T cells. A luciferase assay was performed to detect changes in relative luciferase activity. **E** Co-IP showed the interaction between endogenous WDR4 and Egr1 in UM-UC-3 and 5637 cells. **F** Co-IP showed the interaction between endogenous DDX20 and Egr1 in UM-UC-3 and 5637 cells. **G** IF staining and confocal observation of Egr1, DDX20 and WDR4 in tissue sections from bladder cancer patients. Scale bar 20 μm. **H** Co-IP showed the interaction between endogenous DDX20, WDR4 and Egr1 in WDR4 KD and Control KD UM-UC-3 cells. The data are presented as the means ± SDs, ns, not significant, *p < 0.05, **p < 0.01, ***p < 0.001.

Although WDR4 and DDX20 can bind to the transcriptional regulatory region of ARRB2, neither is an ARRB2 transcription factor. We hypothesized that the WDR4-DDX20 complex may affect the function of specific transcription factors to suppress ARRB2 expression. Transcription factors upstream of ARRB2 were predicted. The results of a yeast two-hybrid assay revealed a possible interaction between DDX20 and Early growth response 1 (Egr1) [29]. Interestingly, the transcription factor Egr1 has 3 binding sites in the ARRB2 promoter (Fig. 6A). These results suggested that the WDR4-DDX20 complex may regulate ARRB2 transcription by inhibiting the function of Egr1. Knockdown of Egr1 markedly decreased ARRB2 expression, whereas overexpression of Egr1 in cells dramatically increased the expression of ARRB2 (Fig. 6B, C, Supplementary Fig. S4E, F). Subsequently, wild-type and mutant plasmids containing these three unique promoter sites were cotransfected individually with the Egr1-overexpressing plasmid in 293 T cells. By the reporter assay, the key binding site of Egr1 in the ARRB2 promoter was determined to be M1 (Fig. 6A, D).

As a scaffold protein, WDR4 promotes the binding of different proteins [17]. Furthermore, the results of co-IP experiments revealed that both WDR4 and DDX20 pulled down Egr1 from the cell lysate, suggesting the existence of a complex consisting of WDR4, DDX20 and Egr1 (Fig. 6E, F). Immunofluorescence analysis by confocal fluorescence microscopy also confirmed that WDR4 colocalized with DDX20 and Egr1 in the nucleus (Fig. 6G). Notably, the association between DDX20 and Egr1 was weaker when WDR4 was knocked down (Fig. 6H). These results suggested that WDR4 can promote the interaction between DDX20 and Egr1. We also found that WDR4 overexpression clearly caused nuclear translocation of DDX20 (Fig. 4N). Given the above results, we hypothesized that WDR4 may mediate the assembly of the DDX20-WDR4-Egr1 complex by recruiting DDX20 to the transcriptional regulatory region of the ARRB2 gene, thereby inhibiting the Egr1-facilitated transcriptional expression of ARRB2.

### WDR4 expression positively correlates with DDX20 expression in bladder cancer and predicts LN metastasis

**Fig. 7:**
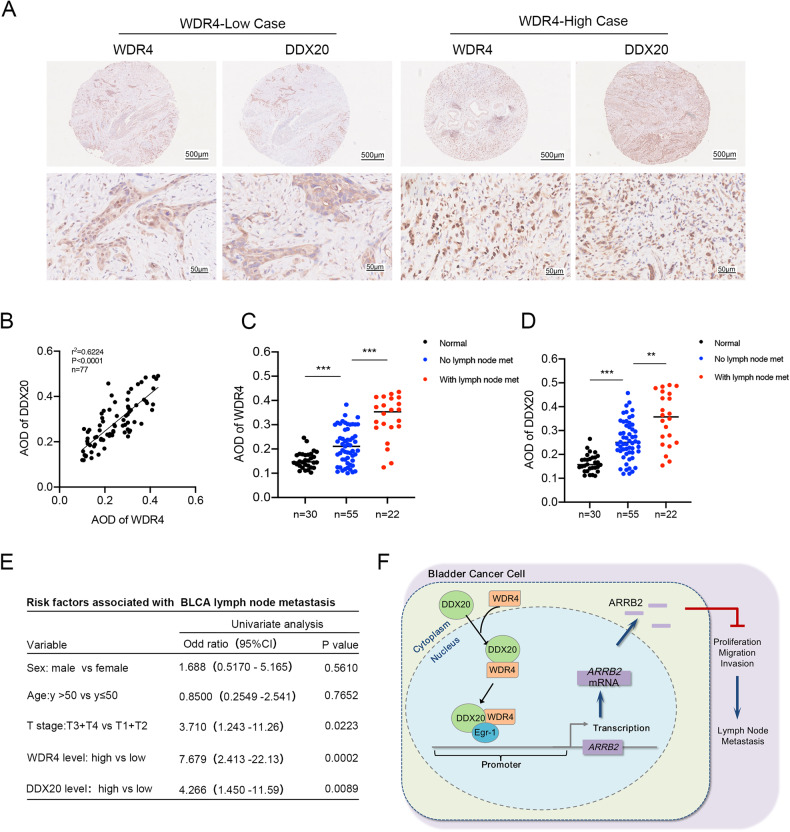
WDR4 expression positively correlates with DDX20 expression and predicts LN metastasis of bladder cancer. **A** Representative images of WDR4 and DDX20 IHC staining in bladder cancer tissues, Scale bar of upper panel = 500 μm, Scale bar of lower panel = 50 μm. **B** Pearson correlation between WDR4 and DDX20 levels in clinical bladder cancer tissues. AOD of WDR4 (**C**) and DDX20 (**D**) in normal bladder tissues and bladder cancer tissues from patients with and without LN metastasis. **E** Univariate analyses to determine risk factors associated with LN metastasis in bladder cancer patients (*n* = 77). **F** Schematic illustration of the mechanism of WDR4 in promoting LN metastasis of bladder cancer.

To investigate the clinical significance of WDR4 and DDX20, we assembled a cohort of 77 bladder cancer tissues and 30 normal adjacent tissues. To evaluate the relationship between WDR4 and DDX20 expression, we performed IHC analysis. Staining of two tissue sections from the same patient with either an anti-WDR4 or an anti-DDX20 antibody showed increased regional WDR4 and DDX20 levels in cancer tissues and a positive correlation between WDR4 and DDX20 expression (Fig. 7A, B). Histological staining showed that WDR4 and DDX20 were both upregulated in cancer tissues compared with normal tissues (Fig. 7C, D). In addition, bladder cancer tissues of patients with LN metastasis displayed markedly higher WDR4 and DDX20 levels than those without LN metastasis (Fig. 7C, D). Furthermore, to assess the predictive value of WDR4 for LN metastasis of bladder cancer, we analyzed clinical information from patients. Univariate logistic regression analysis was performed. The results revealed that the expression of either WDR4 or DDX20, similar to the correlations with T stage, was an independent predictor of LN metastasis in bladder cancer (Fig. 7E). These data confirmed the positive correlation between WDR4 and DDX20 expression and the role of these proteins in regulating LN metastasis, as shown in the schematic (Fig. 7F). The WDR4 expression level may serve as an independent prognostic factor for LN metastasis in bladder cancer patients.

## Discussion

The molecular mechanism of LN metastasis in bladder cancer is largely unknown. WDR4 is associated with various biological processes in tumors. This study found that WDR4 was highly expressed in bladder cancer and was correlated with metastasis and progression. Unlike previous studies of the role of WDR4 in the tumor cell cytoplasm, our study unexpectedly showed a significant increase in the nuclear expression of WDR4 as malignancy increased in bladder cancer. Given these observations, exploring the role of nuclear WDR4 in bladder cancer is a critical need. A subsequent mechanistic study revealed that WDR4 promotes the nuclear translocation of DDX20. As an adaptor, WDR4 binds DDX20 and represses the transcription factor Egr1, thereby inhibiting the transcriptional expression of ARRB2 and ultimately promoting the progression of bladder cancer.

The function and mechanism of WDR4 in the nucleus of tumor cells has not been investigated. Previous studies have shown that WDR4 regulates the translation or degradation of tumor-related proteins in the cytoplasm after binding to interacting proteins. WDR4 plays different roles in various types of tumors. The methyltransferase complex formed by WDR4 and METTL1 promotes the translation of the target mRNAs and promotes tumor progression [12, 13]. WDR4 interacts with EIF2A to promote the translation of CCNB1, thereby promoting the proliferation, metastasis and sorafenib resistance of hepatocellular carcinoma cells [16]. In addition, WDR4 can act as a ubiquitinated substrate adaptor molecule and mediate the degradation of PML by ubiquitination to promote lung cancer progression [17]. We found that WDR4 was highly expressed in bladder cancer tissues and was associated with prognosis (Fig. 1). Notably, the expression of WDR4 in the nucleus was significantly higher than that in the cytoplasm in bladder cancer tissue samples, as determined by IHC and IF staining (Figs. 1G–J and 6G). This suggests that WDR4 may regulate tumor progression through another intranuclear mechanism in bladder cancer. One study reported that WDR4 can preserve DNA integrity during DNA replication [31]. Mechanistic studies revealed that WDR4 interacted with the transcriptional repressor DDX20 and regulated the transcriptional expression of ARRB2 (Fig. 6). For the first time, we found a nuclear role of WDR4 in a cancer, and we found that WDR4 can affect the progression of bladder cancer through transcriptional regulation.

As a member of the WD repeat family, WDR4 has a central peptide-binding pocket that provides the structural basis for protein interactions [32]. This structure facilitates the formation of heterotrimeric or multiprotein complexes involved in the regulation of a wide range of signaling pathway activity and gene expression [33, 34]. We demonstrated that WDR4 can promote the interaction between DDX20 and Egr1 (Fig. 6H). WDR5, another member of the WD repeat family, has also been found to play a role in the LN metastasis process in bladder cancer. WDR5 can epigenetically upregulate the expression of VEGF-C by directly binding to BLACAT2, thereby promoting lymphangiogenesis and lymphatic metastasis [9]. HSF1 promotes multiple steps in the process of LN metastasis in bladder cancer through a novel PRMT5-WDR5-dependent transcriptional mechanism [35]. Recently, OICR-9429, a small molecule inhibitor specifically targeting WDR5, was shown to inhibit the proliferation and reduce the chemosensitivity of bladder cancer cells [36]. In our mass spectrometry analysis, the level of WDR5 in bladder cancer tissues was also found to be increased, but this increase was not as great as the fold increase in the WDR4 level (Supplementary Table S1). This suggests that WDR4-targeted bladder cancer therapy may be developed in the future. The WD-40 repeat (WDR) domain commonly has physicochemical characteristics of high-affinity binding to small molecule drugs and can be a novel target class for drug discovery [19]. Although this research is at an early stage and these results have not yet been clinically validated, WDR proteins are involved in a variety of disease-related pathways, and the development of new drugs targeting this domain is promising.

How WDR4 is involved in LN metastasis and bladder cancer progression remains a crucial question. We found through an IP experiment that WDR4 bound the transcription suppressor DDX20 (Fig. 4A). The C-terminus of DDX20 has intrinsic transcriptional inhibitory activity and inhibits SF-1 activation [27]. In addition, DDX20 can precipitate the histone deacetylases HDAC2 and HDAC5; thus, it possibly inhibits transcription through recruitment of HDACs [28]. DDX20 is a DEAD-box RNA helicase involved in multiple cellular processes. In metastatic breast cancer, increased DDX20 levels were correlated with patient survival [37]. DDX20 levels were also increased in prostate and colorectal cancers with distant metastases [38]. This suggests that DDX20 may play a role in promoting cancer metastasis. One study reported that the DDX20 gene variant was a potential predictive marker for NMIBC treatment and clinical outcomes [39]. Our results also demonstrated that DDX20 promoted the metastasis and proliferation of bladder cancer cells (Fig. 4D–H). Moreover, the CUT&Tag results showed that DDX20 binds to many gene transcriptional regulatory regions in bladder cancer cells (Fig. 5B).

We found that WDR4 and DDX20 bind simultaneously to the target gene ARRB2 (Fig. S4D). ARRB2 is a classical attenuator of G-protein-coupled receptors. Downregulation of ARRB2 promotes lung and prostate cancer growth [40, 41]. One study found that ARRB2 is a potential prognostic indicator for progression and the chemotherapy response in bladder cancer, and overexpression of ARRB2 leads to reduced tumor growth and an enhanced response to chemotherapy [30]. Our study demonstrated that ARRB2 inhibited the metastasis and proliferation of bladder cancer cells (Fig. 5I–M). The above results indicated that ARRB2 has an inhibitory effect on bladder cancer. Subsequently, three binding sites of the Egr1 transcription factor in the promoter of ARRB2 were predicted and confirmed (Fig. 6A, D). Egr1 is significantly associated with human cancer and plays a role in the proliferation, apoptosis, migration, and invasion of cancer cells and in the tumor microenvironment. DDX20 has been found to interact with Egr2 and has been shown to inhibit transcriptional activation mediated by Egr2. Egr2 is an important paralog of Egr1. A mammalian two-hybrid experiment showed that DDX20 could also interact with Egr1 [29]. WDR4 can facilitate the binding of DDX20 and Egr1 (Fig. 6H). This study demonstrated that WDR4 modulates ARRB2 transcriptional expression by promoting DDX20 translocation into the nucleus and inhibiting Egr1 activation.

In summary, we found for the first time that WDR4 is highly expressed in the nucleus of cancer cells and promotes LN metastasis and progression in bladder cancer. A new regulatory mechanism was revealed in which WDR4 acts as an adaptor to promote transcriptional inhibition of tumor suppressor genes. Moreover, WDR4 is a promising target for molecular targeted therapy and drug development. The results of this study are anticipated to facilitate the delineation of the mechanisms bladder cancer progression to support the prospective exploration of precise and effective clinical treatments.

## Materials and methods

### Label-free quantitative proteomic analysis

Bladder cancer tissues (T) and paired normal adjacent tissues (N) for proteomic analysis were obtained from patients undergoing surgery at Nanjing Drum Tower Hospital. The study was approved by the Ethics Committee of Nanjing Drum Tower Hospital (project number: 2018-015-01). Fresh tissues from 17 patients were divided into two batches for label-free proteomic analysis performed by Jingjie PTM Biolab (Hangzhou, China). The workflow included protein extraction and digestion, high-performance liquid chromatography (HPLC) fractionation, liquid chromatography–tandem mass spectrometry (LC‒MS/MS), and bioinformatic analysis. Proteins with a fold change in expression of > 1.5 and a *p* value of < 0.05 were considered to be significantly differentially expressed. Differentially expressed proteins were determined by taking the intersection of the two proteomic datasets.

### Cell culture

Human bladder cancer cell lines (UM-UC-3, 5637, T24, and J82), the ureteral epithelial immortalized cell line SV-HUC-1, and the embryonic kidney cell line 293 T were obtained from the Cell Bank of the Chinese Academy of Science (Shanghai, China). UM-UC-3, J82 and 293 T cells were cultured in Dulbecco’s modified Eagle’s medium (DMEM), T24 cells were cultured in McCoy’s 5 A medium, 5637 cells were cultured in RPMI 1640 medium, and SV-HUC-1 cells were cultured in F-12K medium under standard procedures and maintained at 37 °C with 5% CO_2_. All complete media were supplemented with 10% (v/v) fetal bovine serum (FBS) (Gibco, USA), 100 U/mL penicillin, and 100 µg/ml streptomycin (Wisent, China).

### RNA interference, plasmids and lentiviruses

Small interfering RNAs (siRNAs) against human WDR4, DDX20, Egr1 and ARRB2 were obtained from Generay (Shanghai, China). The siRNAs are listed in Supplementary Table S7. Cells were transiently transfected with siRNAs against the target genes and with the negative control siRNA using jetPRIME (Polyplus, France). The WDR4 shRNA, TET-inducible Flag-WDR4, and corresponding control lentiviruses were obtained from GeneChem (Shanghai, China). Cells were infected with lentivirus, and puromycin was used to select surviving positively transduced cells. The FLAG-tagged Egr1-overexpressing plasmid and the empty vector (pcDNA3.1-3xFlag-C) were purchased from Youbio (Changsha, China). The reporter plasmids containing the wild-type (WT) and mutant (MUT) promoters of ARRB2 were purchased from Tsingke (Beijing, China). Plasmid transfection was performed with Lipofectamine 3000 (Invitrogen). The luciferase assay was performed using the Dual Luciferase Reporter Assay System (Promega).

### Real-time quantitative polymerase chain reaction (RT‒qPCR)

Total RNA was extracted, and cDNA was synthesized. RT‒qPCR was performed using ChamQ Universal SYBR qPCR Master Mix (Vazyme, China). Data were acquired with a QuantStudio 6 Flex PCR System (Thermo Fisher, USA). Fold changes in mRNA levels were calculated using the comparative Ct method (2^−△△CT^). The specific primers are listed in Supplementary Table S7.

### Western blotting (WB)

Protein extracts were prepared on ice using lysis buffer containing proteinase inhibitor. Samples were denatured, and proteins were separated by SDS‒PAGE and then transferred to polyvinylidene difluoride (PVDF) membranes. After blocking, the PVDF membranes were incubated first with primary antibodies and then with secondary antibodies. Antibodies against WDR4 (ab169526) were purchased from Abcam (Cambridge, MA, USA), and antibodies against DDX20 (11324-1-AP), Egr1 (22008-1-AP), and ARRB2 (10171-1-AP) were purchased from Proteintech (Rosemont, IL, USA). Signals were detected using an enhanced chemiluminescence (ECL) system (Vazyme, Nanjing, China), and images were acquired by a ChemiScope 3300 Mini Imaging System (CLiNX, Shanghai, China).

### Cell migration and invasion assays

For the wound healing assay, cells were cultured to nearly 100% confluence, and a scratch wound was made in the surface of the cell layer. Cells were maintained in serum-free medium for a specified period of time. After observation and imaging of the cells, the migration distance was measured and calculated. For the Transwell migration assay, cells (6 × 10^4^ cells/well) were seeded in the upper chambers (8 μm) and maintained in serum-free medium, whereas conditioned medium was added to the lower chambers. For the Transwell invasion assay, Matrigel was spread on the bottom surface of each upper chamber and allowed to solidify at 37 °C. Cells were then seeded in the upper chambers for evaluation of invasion. After culture at 37 °C, the cells were fixed, stained and photographed.

### Cell proliferation assay

Cell proliferation activity was evaluated using colony formation and EdU incorporation assays. For the colony formation assay, cells (300 cells/well) were seeded in 12-well plates and cultured for 7 days. The cells were then fixed and subsequently stained with crystal violet. For the EdU incorporation assay, cells (4 × 10^4^ cells/well) were plated in 48-well plates. When the confluence reached 50%, the cells were treated with the components of the BeyoClicK EDU-488 Kit (Beyotime). The cells were imaged, and the numbers of colonies and proliferating cells were determined using ImageJ.

### Immunofluorescence (IF)

Cultured cells were fixed with 4% paraformaldehyde (PFA) and permeabilized with 0.3% Triton X-100. The samples were blocked with 5% BSA and incubated with specific primary antibodies overnight at 4 °C. After rinsing, the samples were incubated with fluorescence-labeled secondary antibodies at RT for 1 h. Nuclei were stained with DAPI. Slides were photographed using EVOS FL Auto 2.0 Imaging System (Thermo Fisher). Nikon Eclipse Ci-S was used for protein colocalization observation.

### immunoprecipitation (IP)

Cells were lysed on ice using lysis buffer containing proteinase inhibitor. Primary antibodies (2 µg per sample) were added for incubation overnight at 4 °C, with immunoglobulin G (IgG) as the control. Antibodies against WDR4 (ab241297) were purchased from Abcam (Cambridge, MA, USA), and antibodies against DDX20 (11324-1-AP) were purchased from Proteintech (Rosemont, IL, USA). Normal rabbit IgG (2729) was purchased from Cell Signaling Technology (Danvers, MA, USA). Protein A/G Magnetic Beads (Cell Signaling Technology) were then added for incubation at RT. Then, immunoprecipitated proteins were separated. Ultimately, the interacting proteins were eluted and sent for analysis by mass spectrometry or WB.

### Cleavage under targets and tagmentation sequencing (CUT&Tag-seq)

WDR4- and DDX20-specific binding DNA fragments were detected using the CUT&Tag Kit (Vazyme). Briefly, cells (1 × 10^5^ per sample) were captured with ConA magnetic beads and sequentially incubated with a specific antibody, secondary antibody and pA-Tn5. Antibodies against WDR4 (ab169526) were purchased from Abcam (Cambridge, MA, USA), and antibodies against DDX20 (11324-1-AP) were purchased from Proteintech (Rosemont, IL, USA). After fragmentation and extraction of DNA, the library was prepared by PCR and then sequenced on the HiSeq platform in PE150 mode. The data were analyzed using a cloud-based bioinformatics platform (Vazyme).

### Hematoxylin-eosin (H&E) and immunohistochemical (IHC) staining

Formalin-fixed tissues were collected and prepared into tissue chips and were then stored in a −20 °C freezer. The slides were incubated at 75 °C, dewaxed in xylene, and then rehydrated. For H&E staining, the slides were stained with hematoxylin for 1 min and eosin for 30 sec. For IHC, the slides were blocked and incubated first with primary antibodies overnight at 4 °C and then with HRP-conjugated secondary antibodies at RT for 1 h. Finally, signals were detected with a diaminobenzidine (DAB) kit. Images were acquired using a NanoZoomer S60 slide scanner (Hamamatsu). Using Image-Pro Plus professional image processing software, the integrated optical density (IOD) and area were measured, and the average optical density (AOD) was calculated as follows: IOD/area. The AOD value was considered an indicator of differences in protein expression.

### Animal experiments

All animal experiments were conducted according to the guidelines of the Institutional Animal Care and Use Committee of Nanjing Drum Tower Hospital. BALB/c male nude mice aged 4-6 weeks were obtained from GemPharmatech (Nanjing, China). For the LN metastasis models, UM-UC-3 cells (3 × 10^6^ per mouse) with stable WDR4 knockdown or overexpression or the corresponding control cells were inoculated into the footpad of each mouse. The primary tumor and popliteal LNs were assessed by in vivo imaging 30 days after cell implantation. For the xenograft models, the abovementioned cells (3 × 10^6^ per mouse) were inoculated subcutaneously in the right flanks of mice. After 4 weeks, the tumors were harvested, weighed and photographed. The tumor volume was calculated using V = 1/2 × L × W^2^, where L is the largest and W is the smallest diameter. After the mice were sacrificed, the popliteal LNs or tumors were excised. The tissues were fixed, paraffin-embedded and analyzed by H&E and IHC staining.

### Statistical analysis

The results are presented as the means ± SDs. Statistical analyses involving two groups were performed using Student’s t test, and analyses involving multiple groups were performed using one-way ANOVA. All data analyses were performed with GraphPad Prism 7 software. Differences with *p* values < 0.05 were considered to be significant.

### Supplementary information

Figure S1

Figure S2

Figure S3

Figure S4

Supplementary data-Supplementary figure legends

Supplementary Table S1

Supplementary Table S2

Supplementary Table S3

Supplementary Table S4

Supplementary Table S5

Supplementary Table S6

Supplementary Table S7

Original Data File

## Data Availability

The datasets used and analyzed during the current study are available from the corresponding author on reasonable request.
