# Supplementary figures and images for: WDR4 promotes the progression and lymphatic metastasis of bladder cancer via transcriptional down-regulation of ARRB2
Source: Oncogenesis. 2023 Oct 2;12(1):47. doi: 10.1038/s41389-023-00493-z (PMC10545698; doi:10.1038/s41389-023-00493-z)

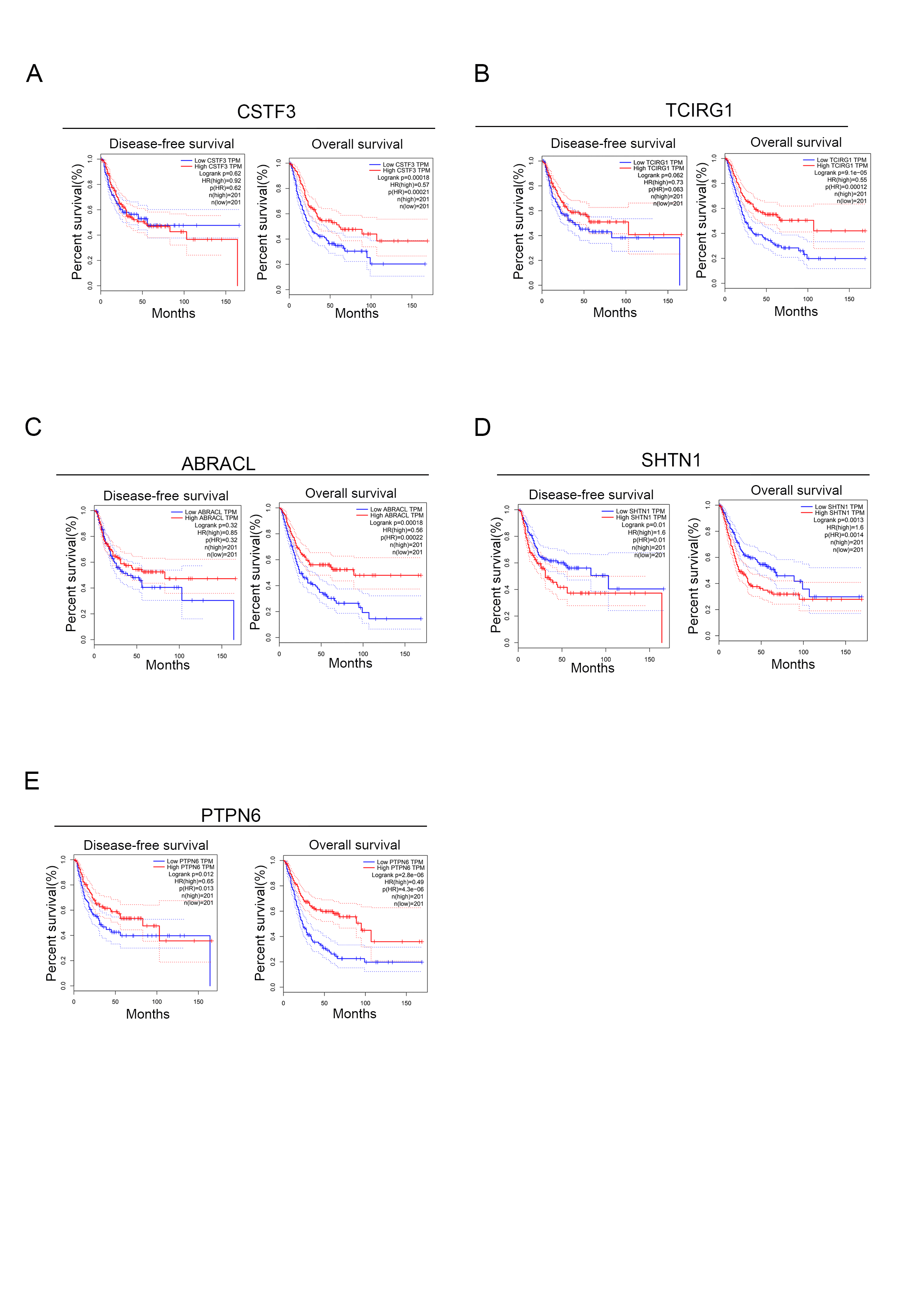

Supplement: Supplementary file 1 — Figure S1 [file 41389_2023_493_MOESM1_ESM.tif]

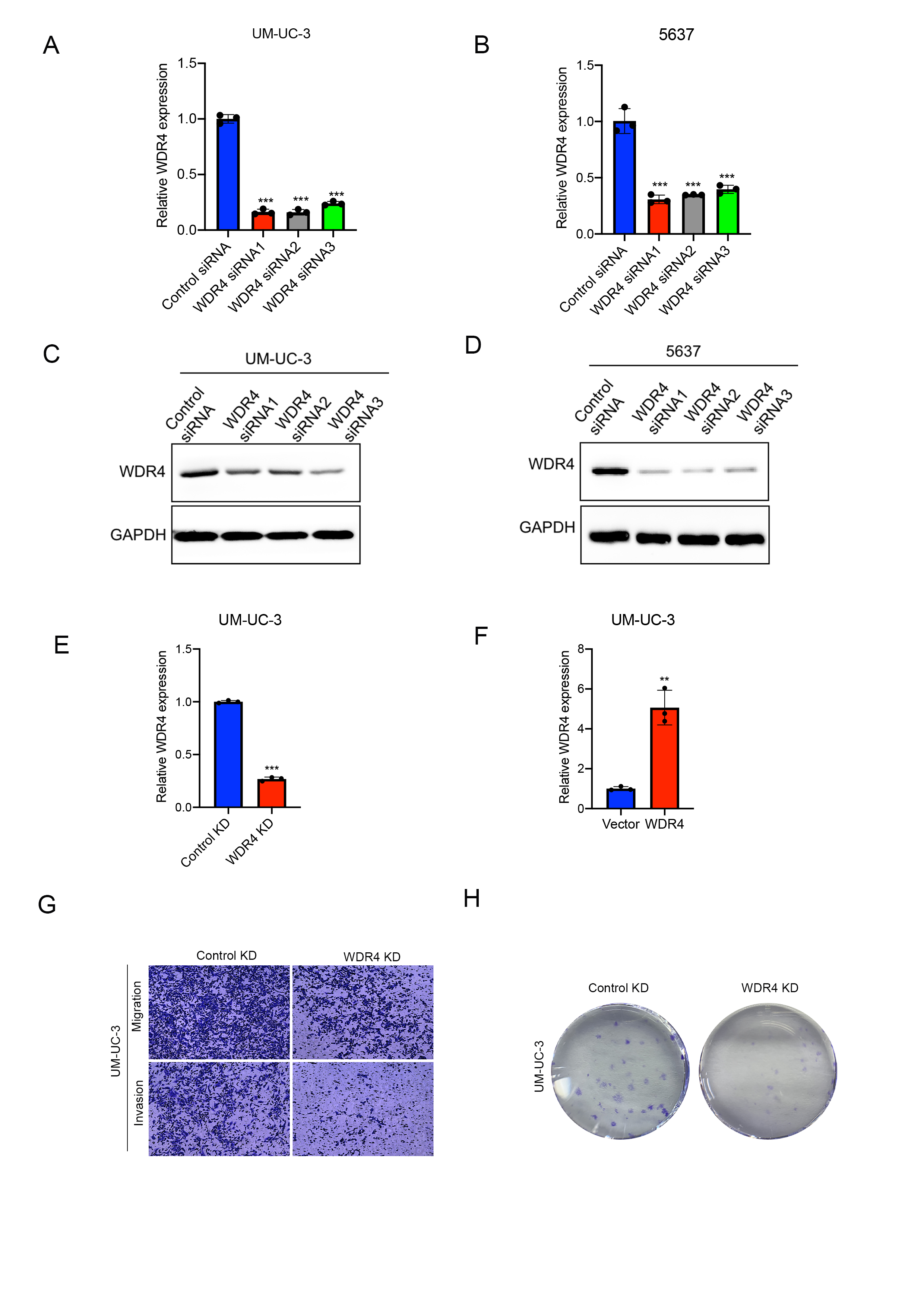

Supplement: Supplementary file 2 — Figure S2 [file 41389_2023_493_MOESM2_ESM.tif]

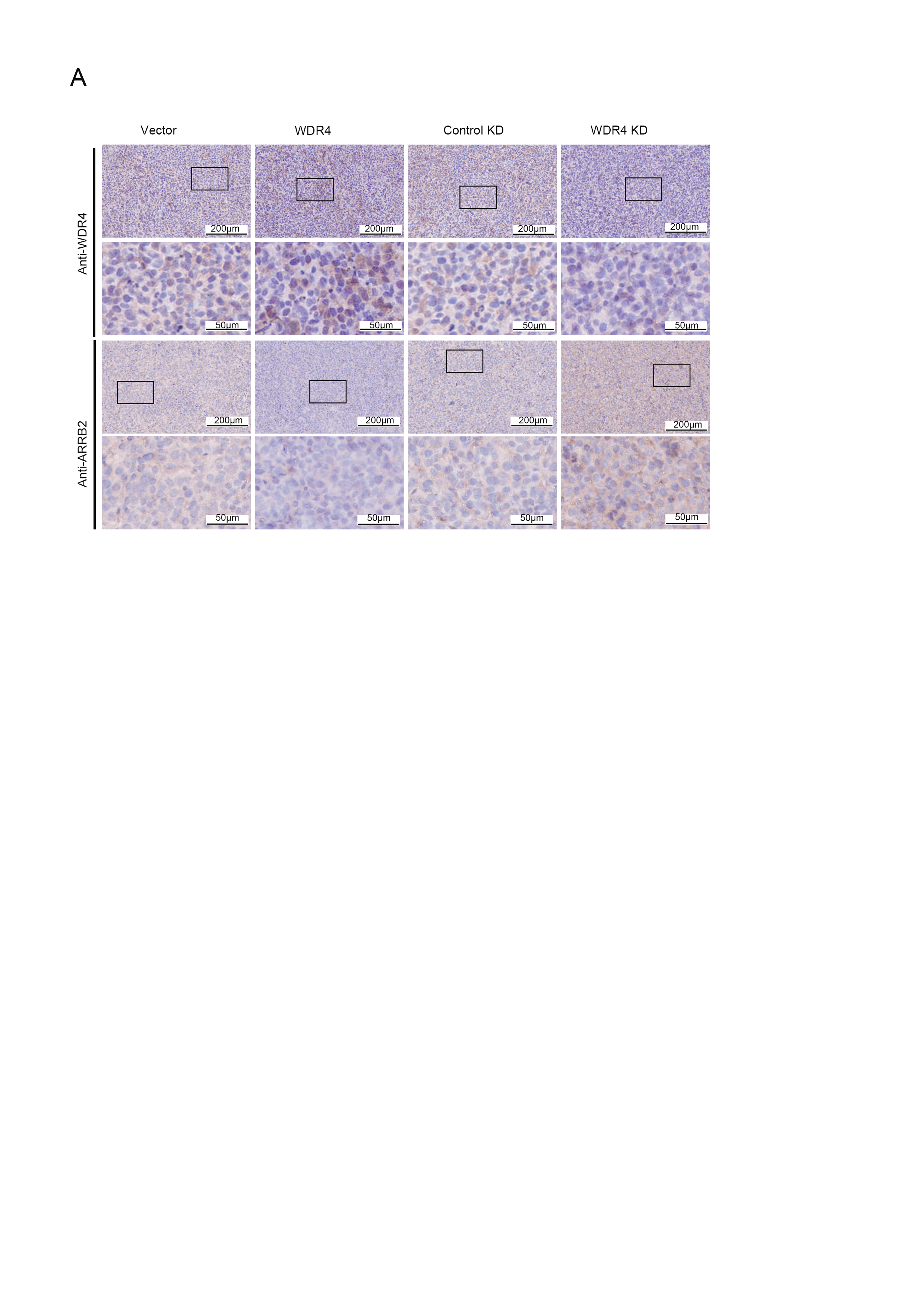

Supplement: Supplementary file 3 — Figure S3 [file 41389_2023_493_MOESM3_ESM.tif]

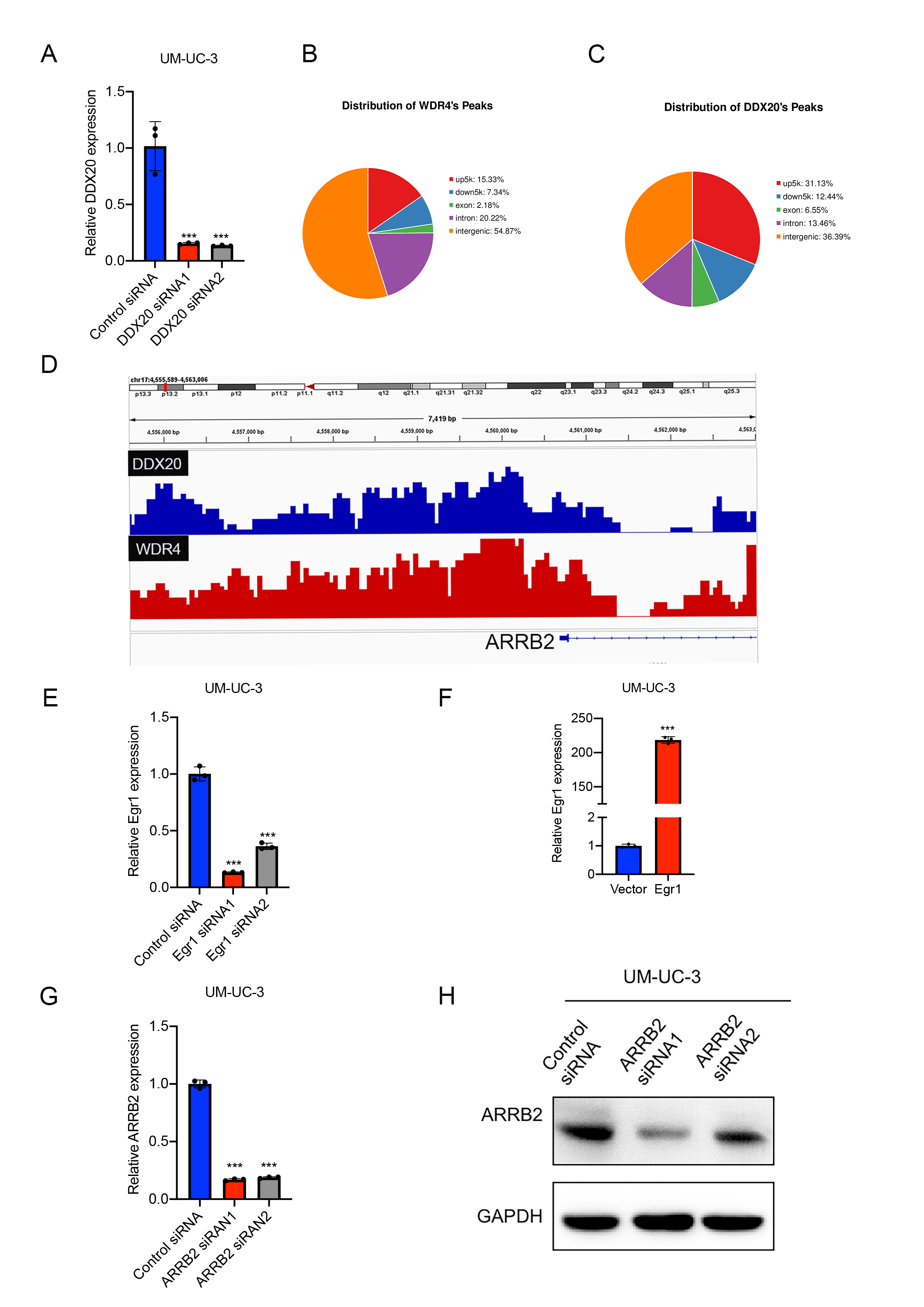

Supplement: Supplementary file 4 — Figure S4 [file 41389_2023_493_MOESM4_ESM.tif]
