# Supplementary material for: WDR4 promotes the progression and lymphatic metastasis of bladder cancer via transcriptional down-regulation of ARRB2
Source: Oncogenesis. 2023 Oct 2;12(1):47. doi: 10.1038/s41389-023-00493-z (PMC10545698; doi:10.1038/s41389-023-00493-z)
Supplement: Supplementary file 5 — Supplementary data-Supplementary figure legends [file 41389_2023_493_MOESM5_ESM.docx]

**Supplementary Figure Legends**

**Fig. S1.** Survival analysis of patients with bladder cancer. Kaplan‒Meier analysis of DFS and OS for bladder cancer patients with low or high levels of CSTF3 (A), TCIRG1 (B), ABRACL (C), SHTN1 (D), and PTPN6 (E) expression in the BLCA cohort obtained from the TCGA database.

**Fig. S2.** Knockdown and overexpression of WDR4. (A-B) UM-UC-3 (A) and 5637 (B) cells were transfected with WDR4-specific siRNAs and the corresponding control siRNA. The mRNA levels of WDR4 in cells were measured by RT‒qPCR. (C-D) WDR4 protein levels in 5637 (C) and UM-UC-3 (D) cells were measured by WB. (E) Stable knockdown (KD) of WDR4 in UM-UC-3 cells was accomplished by using lentiviruses. The mRNA levels of WDR4 were measured by RT‒qPCR. (F) Lentiviruses were also used to construct cells with stable overexpression of WDR4. The efficiency of WDR4 overexpression was validated by RT‒qPCR. The data are presented as the means ± SDs, ***p* < 0.01, ****p* < 0.001. (G-H) Stable knockdown of WDR4 significantly reduced cell migration and invasion in the Transwell assay (G) and suppressed cell proliferation in the colony formation assay (H).

**Fig. S3.** Immunohistochemical analysis of WDR4 and ARRB2 expression in subcutaneous tumors. Stable WDR4-knockdown and WDR4-overexpressing UM-UC-3 cells and the corresponding control cells were used to construct subcutaneous tumor models of bladder cancer in mice. Immunohistochemical (IHC) analysis of xenograft tumors confirmed that WDR4 knockdown resulted in higher expression of ARRB2, but WDR4 overexpression resulted in lower ARRB2 expression. Scale bar of upper panel = 200 μm, Scale bar of lower panel = 50 μm.

**Fig. S4.** WDR4 and DDX20 can bind the transcriptional regulatory region of the ARRB2 gene. (A) UM-UC-3 cells were transfected with DDX20-specific siRNAs and the corresponding control siRNA. The mRNA levels of DDX20 were measured by RT‒qPCR. (B-C) The distribution of WDR4 (B) and DDX20 (C) protein-bound DNA fragments, as determined by CUT&Tag-seq. (D) Image of CUT&Tag-seq results showing the binding of DDX20 and WDR4 proteins to the ARRB2 genomic region. (E) UM-UC-3 cells were transfected with Egr1-specific siRNAs and the corresponding control siRNA. (F) UM-UC-3 cells were transfected with the Egr1 overexpression plasmid and control vector. The mRNA levels of Egr1 were measured by RT‒qPCR. (G-H) UM-UC-3 cells were transfected with ARRB2-specific siRNAs and the corresponding control siRNA. The mRNA levels of ARRB2 were measured by RT‒qPCR (G), and ARRB2 protein levels were measured by WB (H). The data are presented as the means ± SDs, ****p* < 0.001.
