# Supplementary material for: WDR4 promotes the progression and lymphatic metastasis of bladder cancer via transcriptional down-regulation of ARRB2
Source: Oncogenesis. 2023 Oct 2;12(1):47. doi: 10.1038/s41389-023-00493-z (PMC10545698; doi:10.1038/s41389-023-00493-z)

Figure 1E

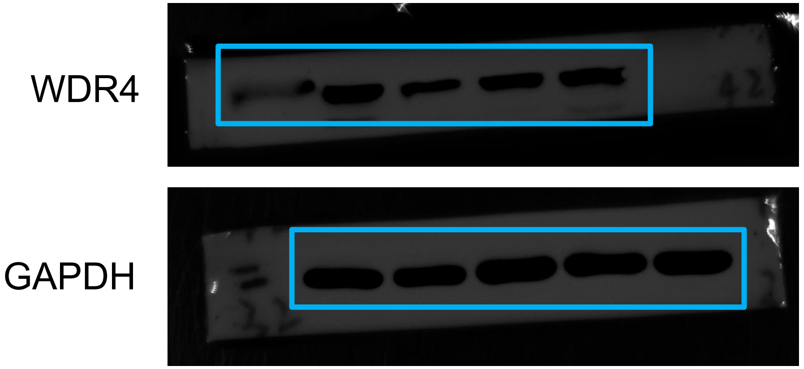

Figure 1F

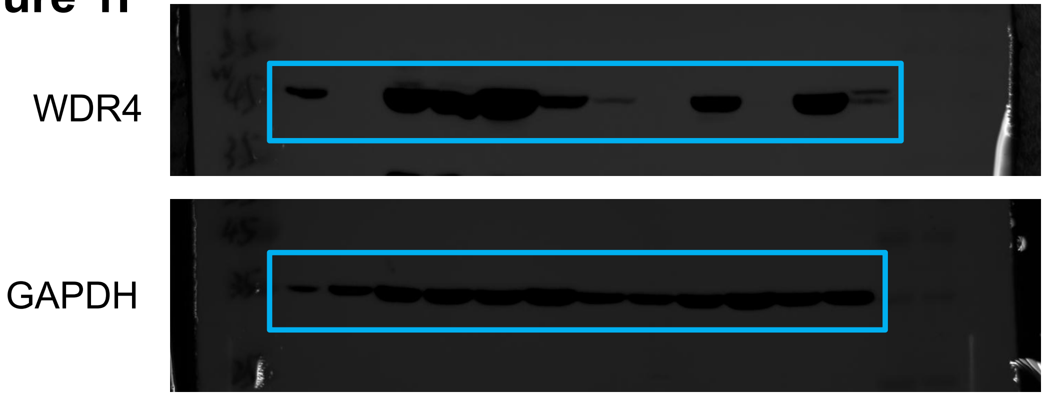

Figure 4B

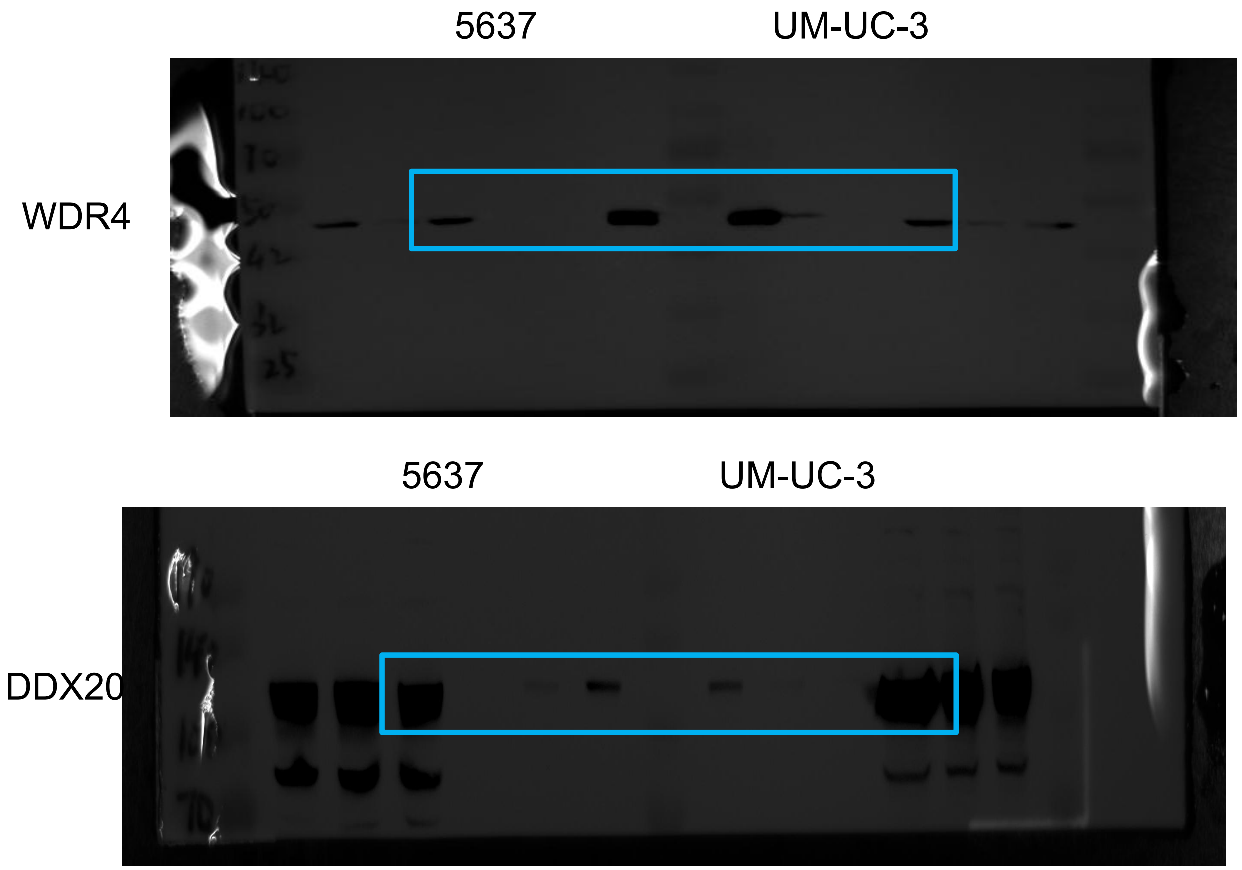

Figure 4C

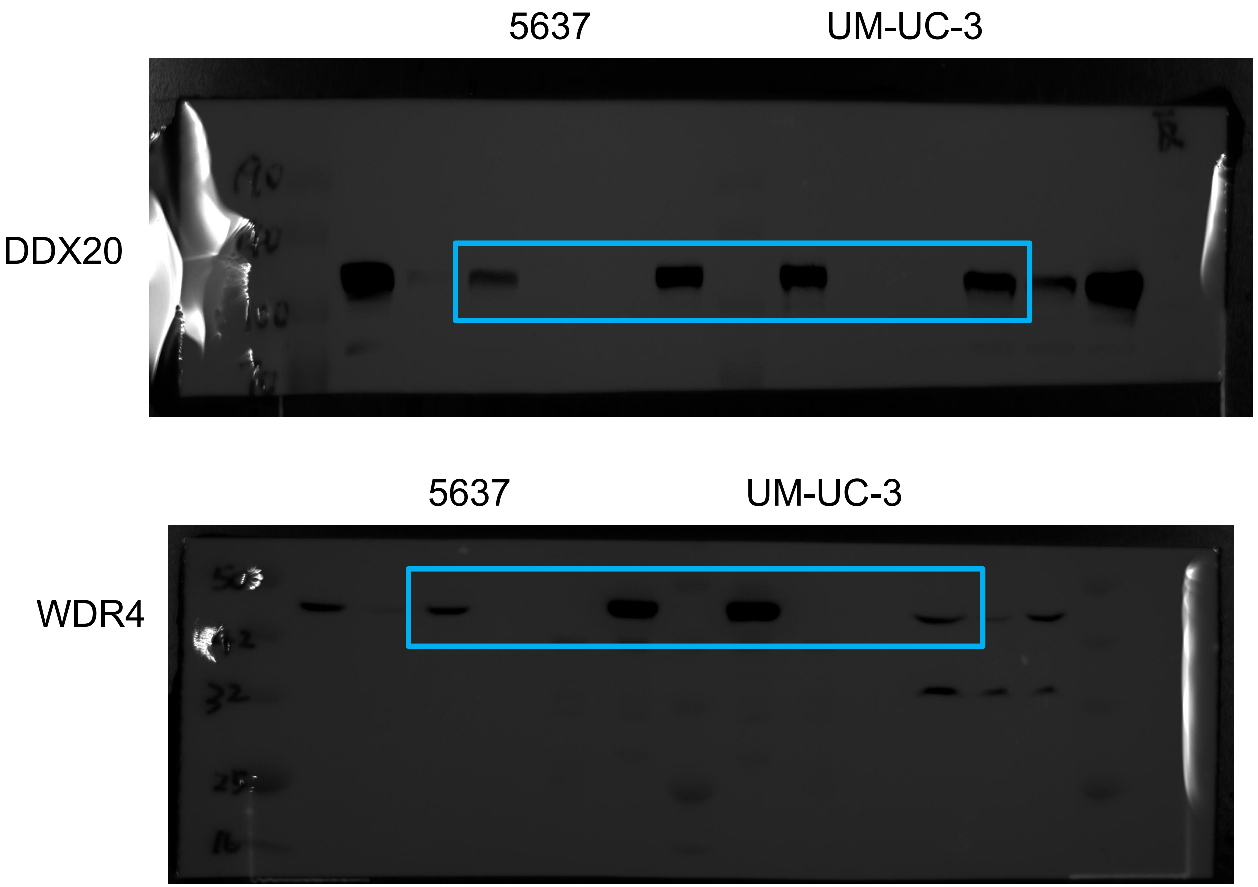

Figure 5E

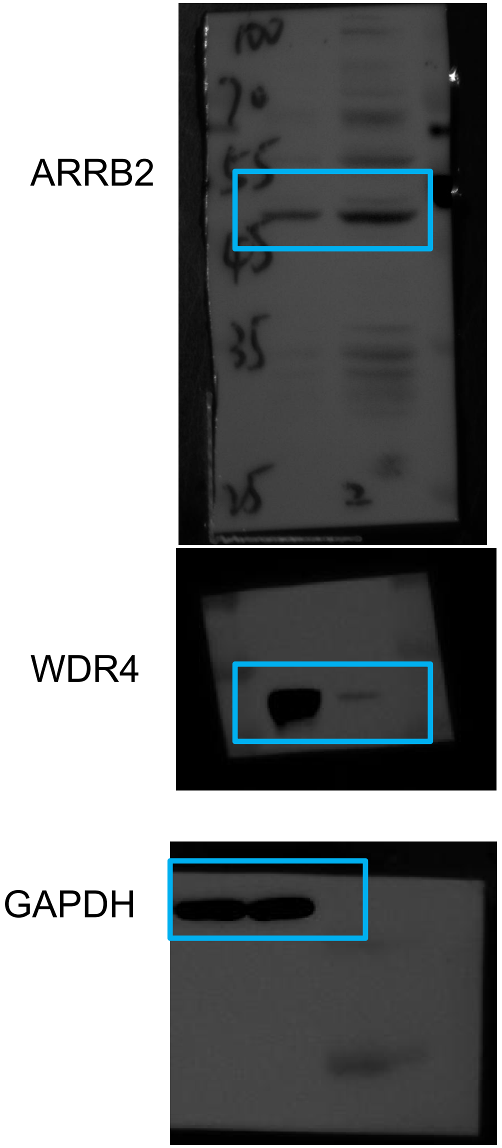

Figure 5F

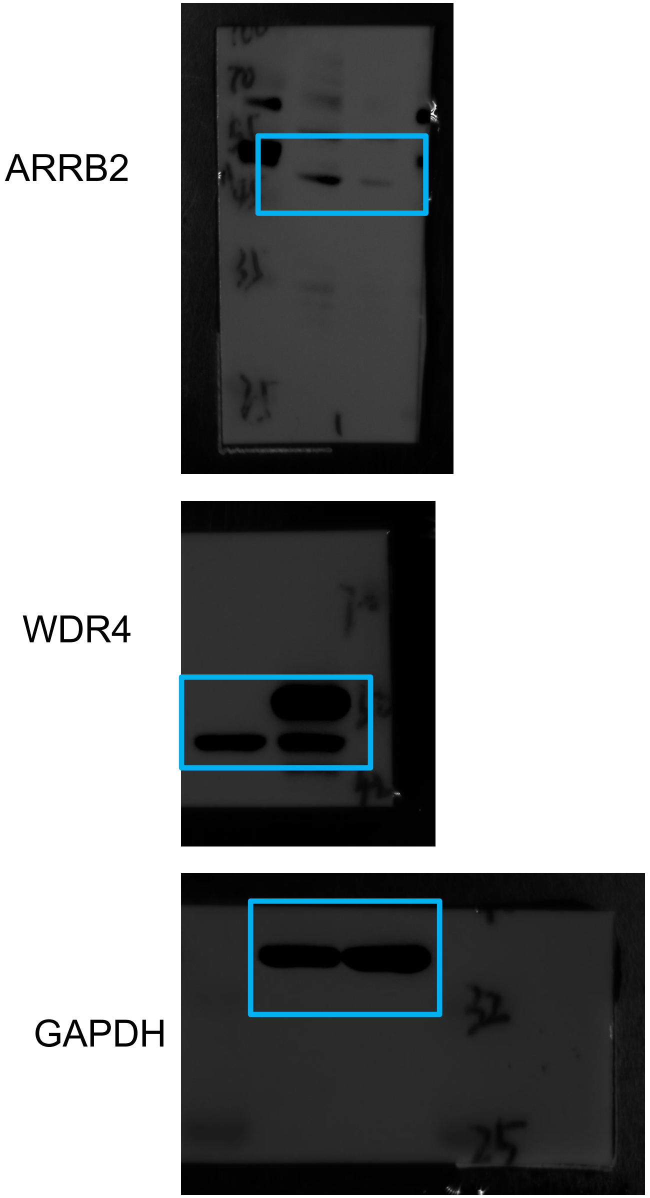

Figure 5H

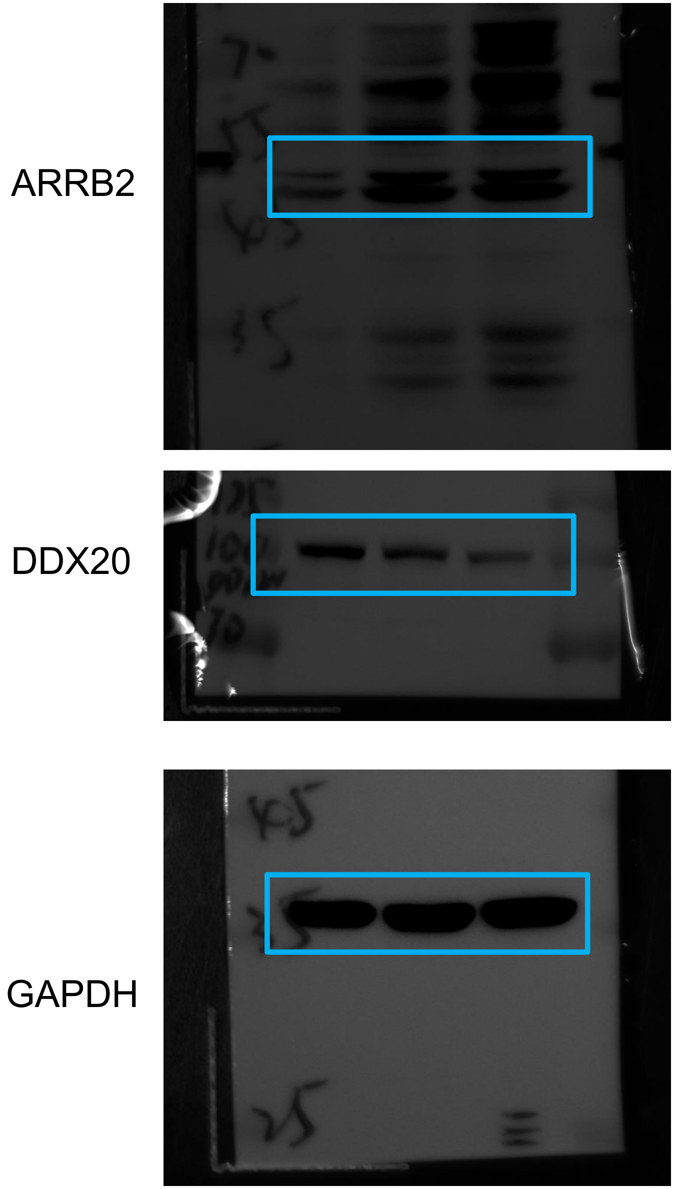

Figure 6C

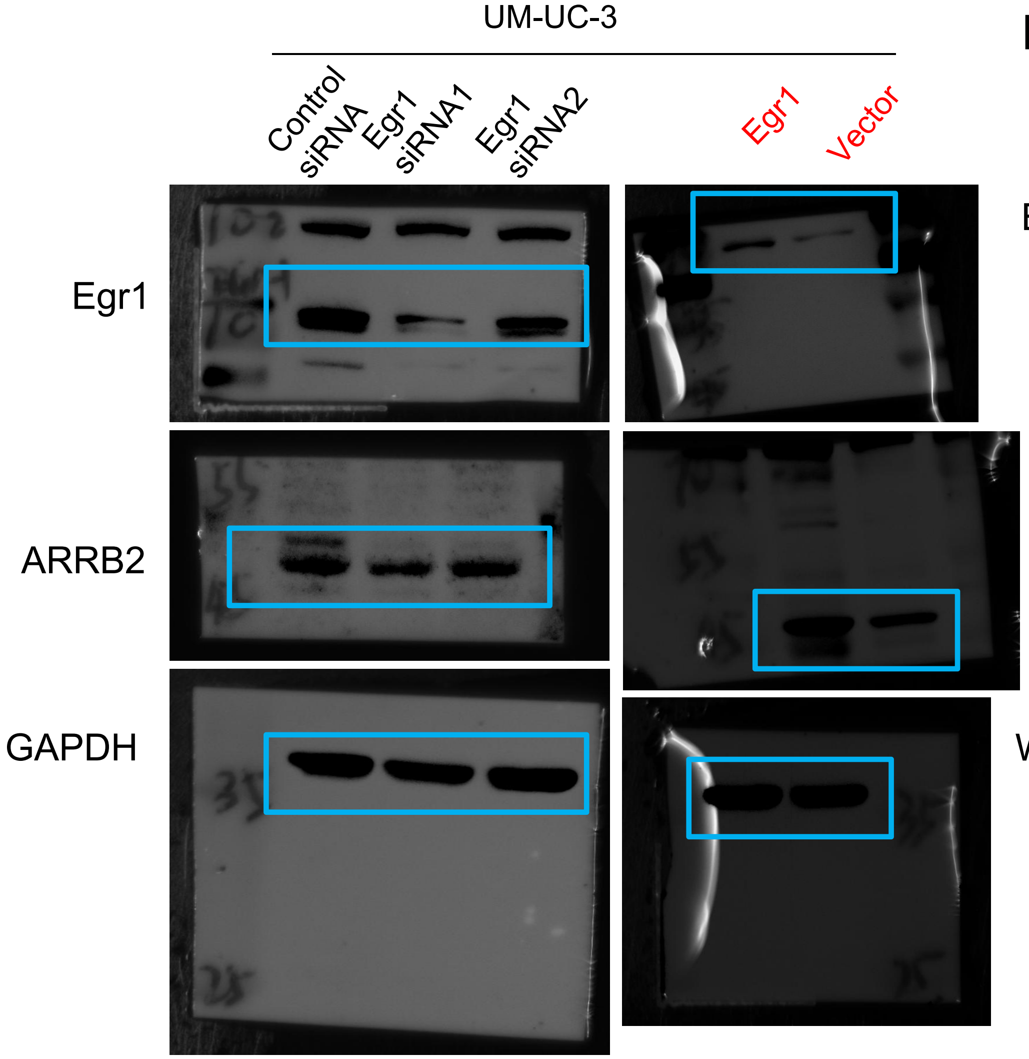

Figure 6E

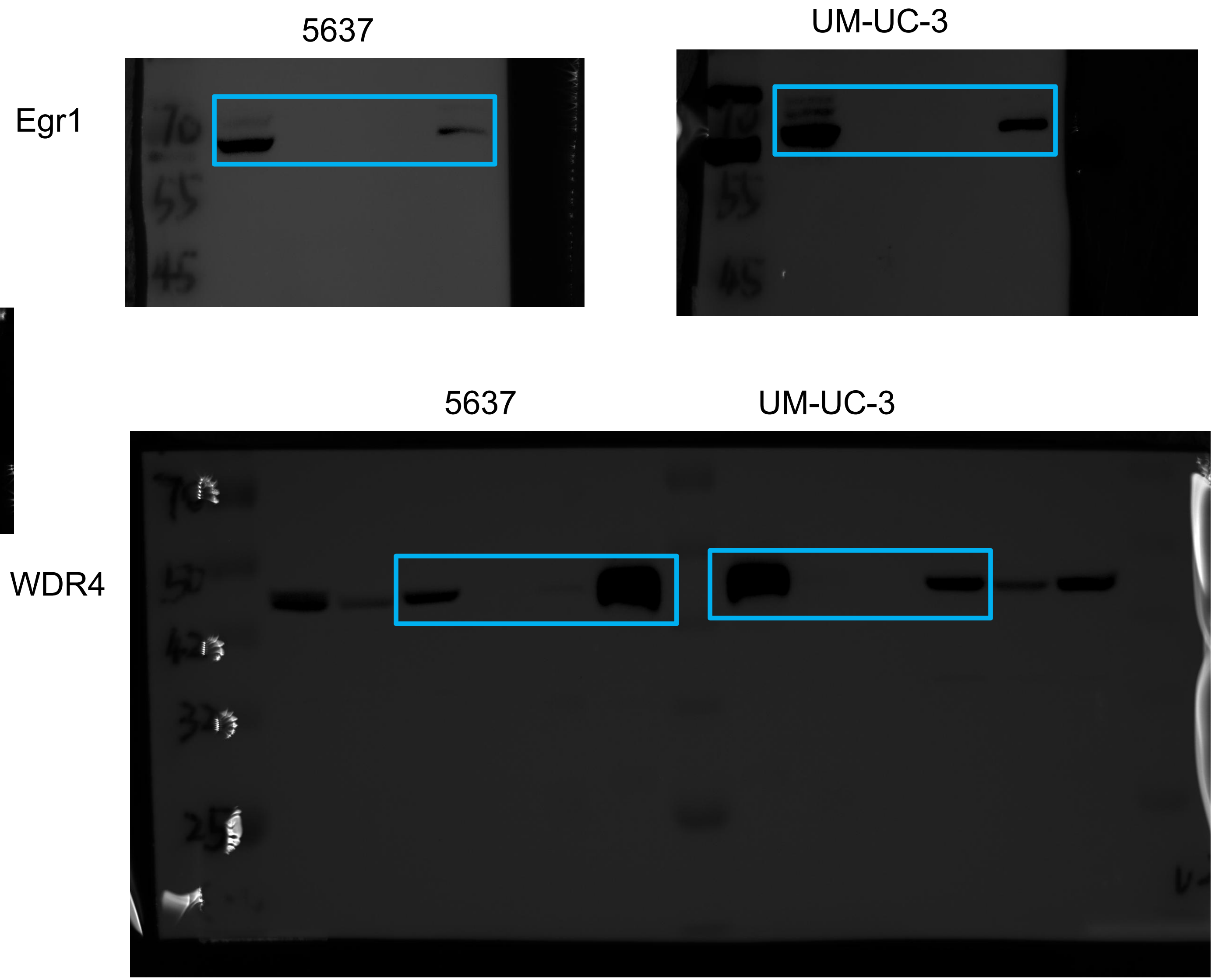

Figure 6F

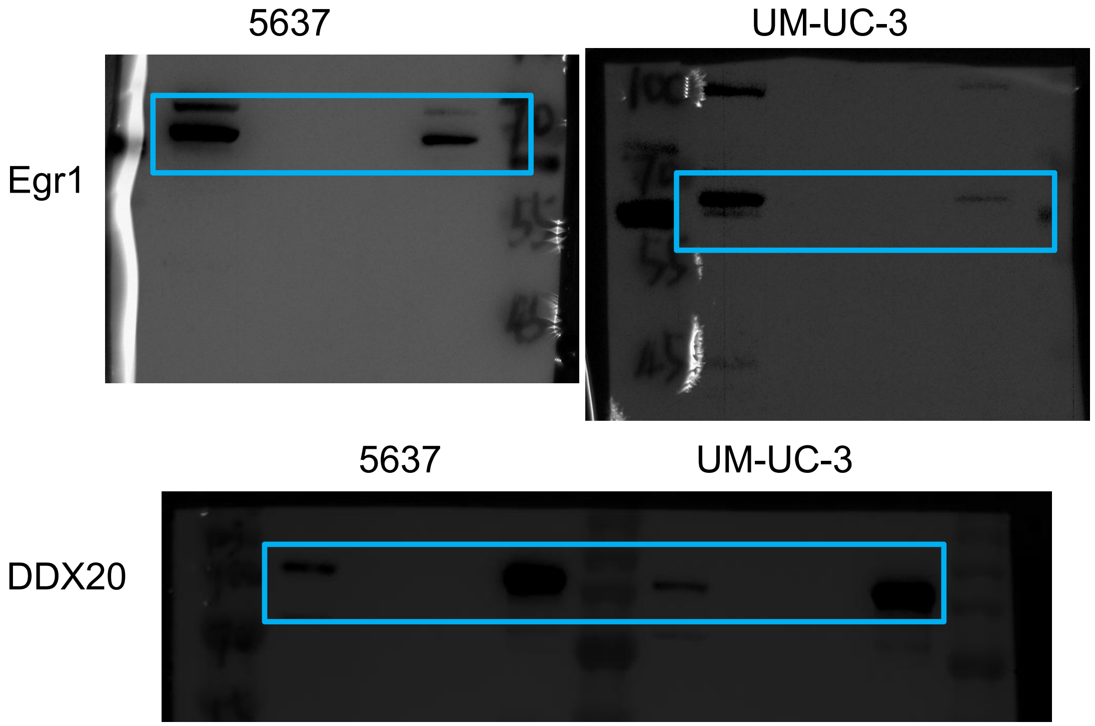

Figure 6H

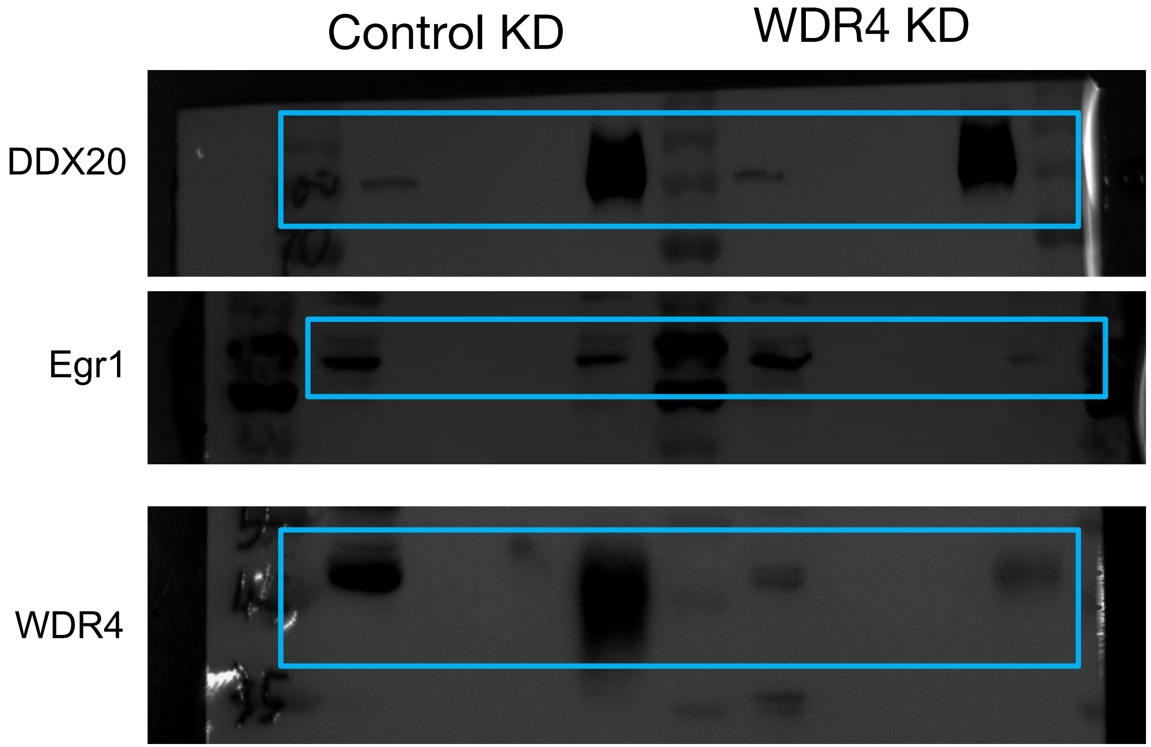

Figure S2C

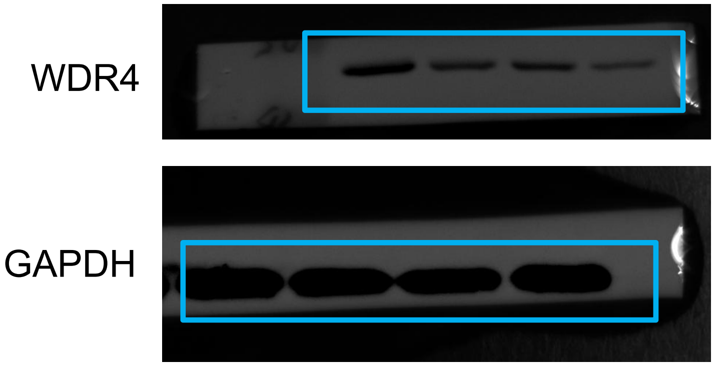

Figure S2D

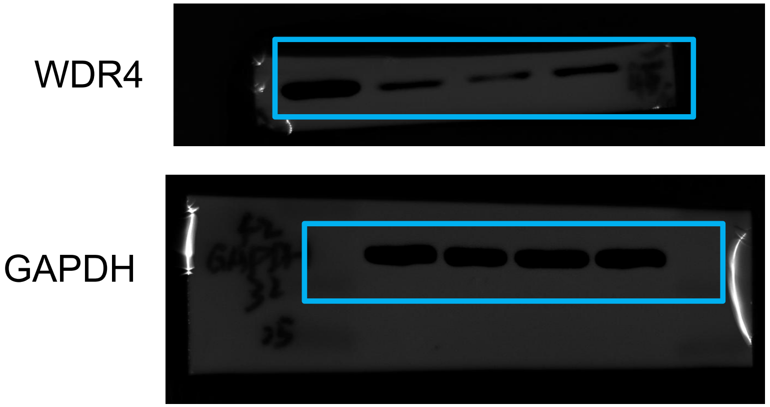

Figure S4H

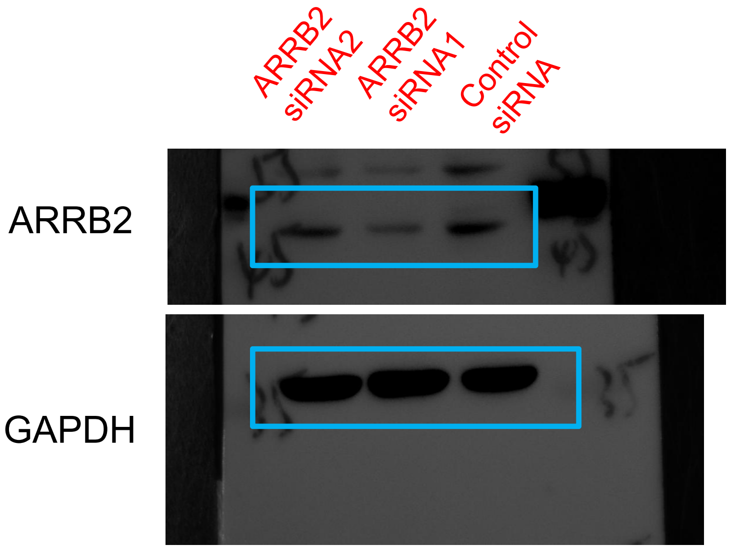

Supplement: Supplementary file 13 — Original Data File [file 41389_2023_493_MOESM13_ESM.pdf]
